# Past and Present Crystallographic Work at the NBS/NIST Reactor

**DOI:** 10.6028/jres.106.046

**Published:** 2001-12-01

**Authors:** A. Santoro

**Affiliations:** National Institute of Standards and Technology, Gaithersburg, MD 20899-8520

**Keywords:** collaborative research, historical research highlights, NBS Reactor, profile refinement, single-crystal and powder neutron diffraction, theoretical crystal geometry

## Abstract

Neutron diffraction at NBS/NIST started soon after the NBS reactor became operational in the summer of 1969. Since that time, literally hundreds of crystal structures have been determined and refined using single crystal and powder neutron diffraction data, collected with a variety of instruments. This work has been usually done in collaboration with other NBS/NIST divisions and/or universities and industrial laboratories. In parallel with the technical developments and the experimental work, also theoretical aspects of crystal geometry have been clarified, and significant improvements in the techniques of profile refinements have been made. It is therefore understandable that a comprehensive description of all the crystallographic studies carried out up to the present is impossible under the constraints of space and time imposed by a review of this type, and, in the following sections, we will limit ourselves to give, only a brief account of the topics which, in our opinion, represent the highlights of the work carried out at the reactor.

## 1. Introduction

Neutron diffraction at NBS/NIST started soon after the NBS reactor became operational in the summer of 1969. Since that time, literally hundreds of crystal structures have been determined and refined using single crystal and powder neutron diffraction data, collected with a variety of instruments. This work has been usually done in collaboration with other NBS/NIST divisions and/or universities and industrial laboratories. In parallel with the technical developments and the experimental work, also theoretical aspects of crystal geometry have been clarified, and significant improvements in the techniques of profile refinements have been made. It is therefore understandable that a comprehensive description of all the crystallographic studies carried out up to the present is impossible under the constraints of space and time imposed by a review of this type, and, in the following sections, we will limit ourselves to give, only a brief account of the topics which, in our opinion, represent the highlights of the work carried out at the reactor.

It is well known that crystal structure analysis provides information, which is fundamental for the understanding of the physical and technological properties of materials. To mention a few examples, drug design would be impossible without the detailed structural knowledge of the complex molecules involved in biological processes, and the assessment of safety margins in metallic parts and components does require measurements of the residual stress done with neutron and x-ray diffraction methods. As described in other chapters of this volume, diffraction measurements are also essential in the study of the magnetic behavior of many materials, and in clarifying how systems such as ice change under the effect of high pressure.

The instrumentation available to the crystallographer today at the NIST reactor must be considered as the best in the United States, and, in particular, the new highresolution neutron powder diffractometer is a world-class instrument whose usefulness will last for many years to come. The crystallographic equipment, coupled with other techniques also available at the reactor, give to the intelligent experimenter the possibility to determine the crystal structure of many materials, even when they can be prepared only in small quantities, and to relate the structural results to those obtained with other techniques.

## 2. Instrumentation

### 2.1 Introduction

By inserting a collimator into the face of a reactor, we can extract a beam essentially consisting of neutrons which have been slowed down by a large number of collisions with the moderator and which are in thermal equilibrium at the reactor’s temperature. The distribution of wavelengths in the collimated beam may be obtained by plotting the function

$$\phi \left(\lambda \right)=\left(const./\lambda^{5}\right)\exp\left[-h^{2}/2mkT\lambda^{2})\right]$$ (1)

as function of *λ* [1]. In this expression, *h* is Plank’s constant (6.625 × 10^−27^ erg s), *k* is Boltzmann’s constant (1.381 × 10^−16^ erg/K), *m* the neutron mass (1.675 × 10^−24^ g) and K the absolute temperature. The function *ϕ* (*λ*) can be defined by saying that *ϕ* (*λ*)d*λ* is the number of neutrons with wavelengths between *λ* and *λ* + d*λ* emerging from the collimator in one second, and its maximum value occurs for the wavelength

$$\lambda =h/{\left(5mkT\lambda^{2}\right)}^{1/2}.$$ (2)

**Fig. 1:**
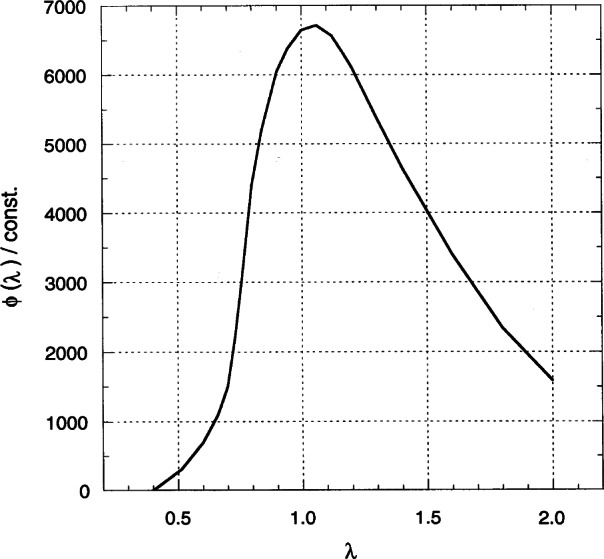
Plot of the function *ϕ* (*λ)/const.* vs *λ* for the reactor equilibrium temperature of 350 K. The product *ϕ(λ)dλ* gives the number of thermal neutrons of wavelength between *λ* and *λ* + d*λ* emerging from an in-pile collimator in one second.

A plot of *ϕ* (*λ*) versus *λ* is shown in Fig. 1 for *T*= 350 K, the equilibrium temperature of our reactor.

**Fig. 2:**
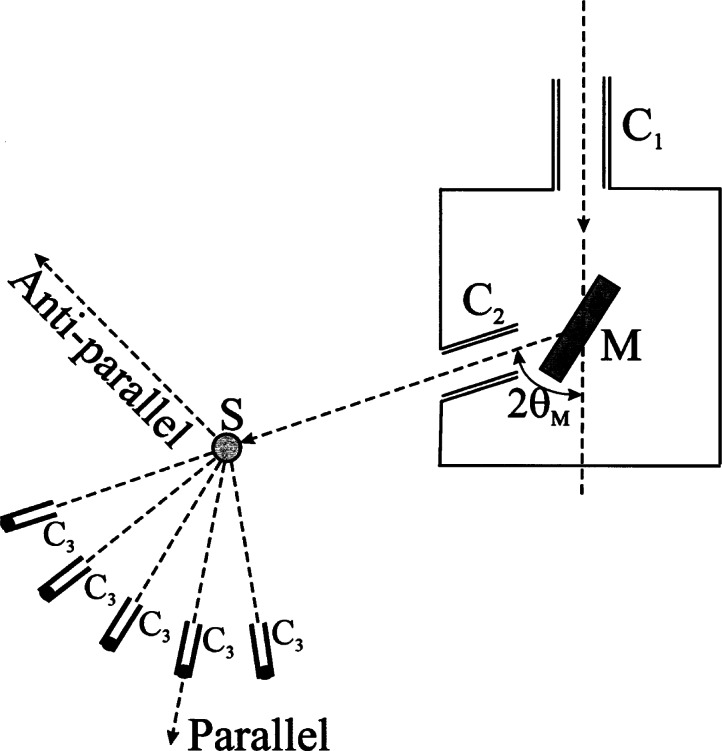
Schematic representation of the first powder diffractometer installed at the BT-1 beam port of the NBS reactor. In its first version, the instrument was equipped with just one counter, which was later replaced by a bank of five detectors with an angular separation of about 20°. In the figure, *C*_1_, *C*_2_ and *C*_3_ represent the in-pile, monochromatic beam and diffracted beam collimators, having angular divergences *a*_1_, *a*_2_ and *a*_3_, respectively. The monochromator M is a single crystal of copper cut with the reflecting surface parallel to the (110) crystallographic planes and oriented to produce the 220 reflection with a take-off angle 2*θ*_m_ of about 75°. With this configuration the monochromatic beam has a wavelength λ of about 1.5 Å. The positions P and A indicate the parallel and antiparallel positions, respectively (see text).

For crystallographic experiments, we must select a narrow band of wavelengths from the beam emerging from the in-pile collimator, and this task is usually accomplished by using as monochromator a single crystal of high reflectivity, cut and oriented so that it will diffract, in the best possible geometric conditions, a beam of wavelengths comprised in a small interval of *λ* centered at

$$\lambda =2d_{m}\sin\theta_{m}$$ (3)

where *d*_m_ is the interplanar spacing of the set of planes involved in the diffraction process, and *2 θ*_m_ is the take-off angle of the monochromator (Fig. 2). The monochromatic beam, after passing through a second collimator, impinges on the sample, is diffracted in a direction which depends on the geometry of the crystal and the technique employed in the experiment, and after passing through a third collimator, is measured by an appropriate detector. The diffracted intensities depend, both for the single crystal and the powder methods, on the structure and the physical state of the sample and on the *luminosity* (i.e., transmission) of the diffractometer used in the experiment. On the other hand, the shapes of the observed peaks are given by the convolution of the *instrumental* profile, due exclusively to the geometrical characteristics of the diffractometer, and the *pure* profile, i.e., the profile that we would observe if the effects of the instrument were negligible. In order to understand the evolution in the design of the diffractometers used at different times at the NBS/NIST reactor, we will briefly discuss the above concepts and their influence on the quality of the collected data.

### 2.2 Instrumental Resolution and Luminosity

Let us assume that the three collimators of a single crystal or powder diffractometer have horizontal angular divergences *α*_1_, *α*_2_ and *α*_3_ (these are defined by the spacing and the length of the Soller slits), and that their transmission function in the horizontal plane of the instrument (which is coincident with the plane of Fig. 2) is Gaussian. Let us also assume that the monochromator is a mosaic crystal with blocks having a Gaussian spread with width at half maximum *β*_m_, and oriented with a take-off angle 2 *θ*_m_. It has been shown [2–4] that, under these assumptions, any diffraction peak centered at the angle *2θ* (where *θ* is the Bragg angle of the peak) has a Gaussian instrumental shape with full width at half maximum *H*_1/2_ given by the equation.

$$H_{1/2}^{2}=U_{0}(\tan^{2}\theta )/\tan^{2}\theta_{m})\pm v_{0}(\tan\theta )/\tan\theta_{m})+W$$ (4)

where *U*_0_ *V*_0_ and *W* are function of the angular divergences *α*_1_, *α*_2_ and *α*_3_ and of *β*_m_ (in the case of single crystal instruments, also the mosaic spread *β*_c_ of the sample must be taken into consideration). Equation (4) is usually written

$$H_{1/2}^{2}=U\tan^{2}\theta \pm V\tan\theta +W$$ (5)

where

$$\begin{array}{cc} U=U_{0}/\tan^{2}\theta_{m}\;\text{and} & V=V_{0}/\tan\theta_{m} \end{array}.$$ (6)

In Eqs. (4) and (5) the plus and minus signs apply to the anti-parallel and parallel positions, respectively, (see Fig. 2) and, since *V* and *V*_0_ are always positive, the value of *H*_1/2_ for the anti-parallel geometry is always greater than that obtained when the peaks are scanned with the parallel configuration. The differences of the full widths at half maximum for the two geometries are significant at all values of *θ* (and have been fully discussed in Refs. [2–4]), and for this reason all two-and three-axes spectrometers are designed to work in the parallel position.

Equation (4) has a minimum at a value of *θ* given by

$$\tan\theta_{\min}=\left(V_{0}/2U_{0}\right)\tan\theta_{m}$$ (7)

with a width

$${\left(H_{1/2}\right)}^{2}=W-\left(V_{0}^{2}/2\;U_{0}\right).$$ (8)

These expressions show that the resolution function of a diffractometer can be controlled, within limits, by choosing appropriately the horizontal divergences of the collimators and the take-off angle of the monochromator. This may not be too important in many cases of single crystal work, where reflections are rarely close enough to one another to cause overlapping, but is essential in the case of the powder method, where serious overcrowding of reflections occurs even for moderately large unit cells.

### 2.3 Powder Diffractometers

The equations discussed before help to explain the evolution in the design of the powder diffractometers at the BT-1 beam port at our reactor. In the initial machine, the monochromatic beam was produced by the 220 reflection of a flat copper monochromator with mosaic spread *β*_m_ of about 15′, oriented with a take-off angle *2 θ*_m_ of about 70°. With this configuration the wavelength of the monochromatic beam is about 1.5 Å, i.e., comparable to the wavelength CuK*_α_*, extensively used in x-ray diffraction experiments. The horizontal divergences of the three Soller collimators were of the order of 20′ and the diffracted intensities were measured using a single detector in the interval 5° ≤ *2θ* ≤ 100°, in steps of 0.05°.

**Fig. 3:**
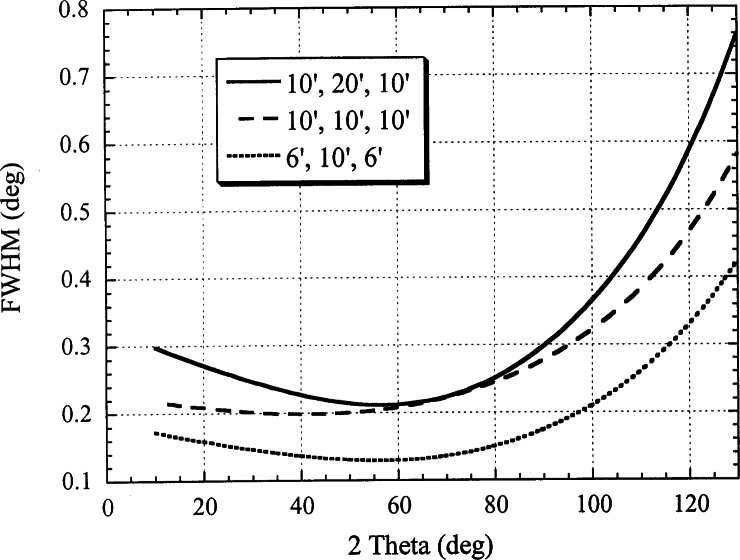
Plot of the full width at half maximum (FWHM) vs *2θ* for three configurations of the in-pile, monochromatic beam and diffracted beam collimators and for the 220 reflection of a copper monochromator of mosaic spread *β*_m_ = 15′ and take-off angle 2*θ*_m_= 75°. The minima of the three resolution functions range from 40° to 60°. The lumininosity of the diffractometer for the 10-10-10 and 6-10-6 configurations is 56 % and 21 % that of the 10-20-10 configuration, respectively.

The advent of the Rietveld method [5,6] in the analysis of crystal structures had the effect of increasing the complexity of the materials that could be studied with the powder method, thus requiring resolutions higher than those obtainable with our original instrument. This was accomplished in the mid seventies by reducing the horizontal divergences of the first and third collimator to about 10′, without changing the basic design of the diffractometer and the take-off angle of the monochromator. The loss of luminosity caused by a collimating system tighter than the one in place before, was more than compensated by replacing the single counter with a bank of five detectors with an angular separation of 20° (Fig. 2). Examples of the resolution obtainable with this instrument are illustrated in Fig. 3 and show that the minima of the resolution functions (i.e., the values of 2*θ* corresponding to the greatest resolution) are all comprised in a region of 2*θ* around 45° to 55°. This is a significant disadvantage because, depending on the unit cell volume and the chemical and physical nature of the sample, severe overcrowding, and consequent overlapping, of the diffraction lines may occur below or above this region of 2*θ.* In these cases it is clearly desirable to have the best instrumental resolution in the angular intervals most affected by overlapping, and Eq. (7) shows that this can be best accomplished by varying the take-off angle of the monochromator. Such change, however, must occur without a significant variation of the wavelength of the monochromatic beam, and with the lowest possible loss of luminosity.

**Fig. 4:**
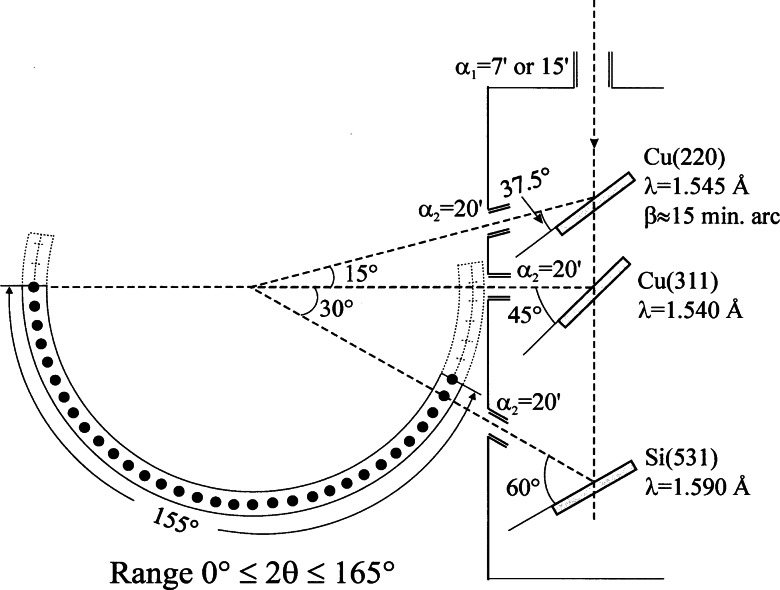
Geometrical features of the new, high-resolution, 32-detector, powder diffractometer now operating at the BT-1 beam port of the NIST Center for Neutron Research reactor. This machine is equipped with three focusing monochromators made of iso-oriented sections, as indicated in the figure, and with take-off angles 2*θ*_m_ equal to 75°, 90° and 120°. The horizontal divergences *a*_1_ and *a*_2_ of the first and second collimators are indicated in the figure, and the divergence *a*_3_ of the third collimator, placed in front of each detector, is 7′. Since the angular separation of successive detectors is 5°, the diffractometer covers a range 0°≤2*θ*≤160° with an excursion of only 5°.

**Fig. 5:**
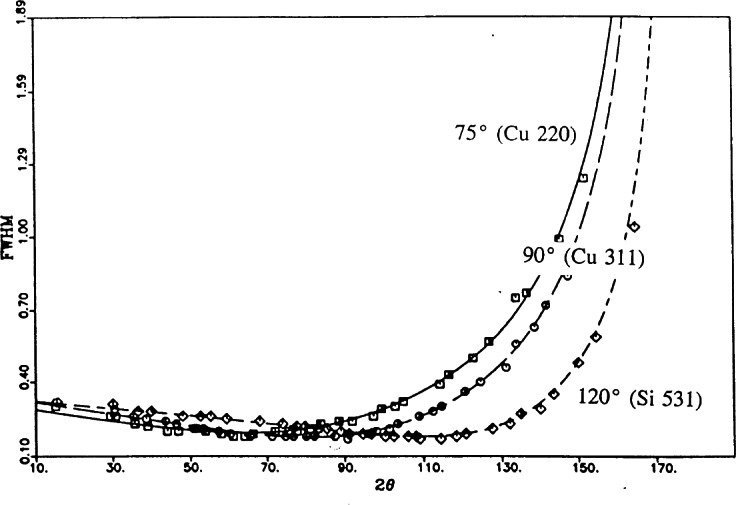
Examples of resolution functions for three monochromators of our new, high-resolution diffractometer. The take-off angle 2*θ*_m_ and the monochromator characteristics are indicated in the figure. It should be noted that, consistently with Eq. (7), the minima of these functions depend on the value of *θ*_m_ and that, in this case, they occur at values of 2*θ*_m_ about equal to the values of 2*θ*_m_ of each function.

The instrument best satisfying these often conflicting requirements was built following a design proposed by E. Prince, of the Reactor Radiation Division, and is schematically illustrated in Fig. 4. Its features have been described in detail [7] and will only be summarized here. With this instrument, the experimenter can measure a powder pattern with any one of three monochromators having take-off angles 2*θ*_m_ equal to 75°, 90° or 120°. The horizontal divergences of the in-pile collimator can be set at 15′ or 7′, while the divergences *α*_2_ are fixed at the values indicated in Fig. 4 and those of the collimators in front of each of the 32 detectors located on the recording arm of the instrument are all equal to 7′. With these conditions, and assuming the mosaic spreads of the monochromators indicated in the figure, one obtains the resolution functions illustrated in Fig. 5. As expected, the minima of these functions occur in different 2*θ* regions. Thus, for the beam generated by the 311 reflection of Ge the minimum is located at 2*θ* ≈ 75° and is mostly used for studying materials with large unit cells and for magnetic materials, since in these cases the intense peaks needed to determine and/or refine the structure are concentrated in the low 2*θ* region of the pattern. On the other extreme, the 531 reflection of the Si monochromator, with its minimum at about 120°, offers high resolution in the region 90° ≤ 2*θ* ≤ 130°, and is most effectively used in those cases in which it is important to detect splits of the diffraction lines (as in the case of phase transitions), or when it is essential to measure with high precision the thermal factors of the atoms in the structure. Finally, the 311 reflection of copper is a compromise between the two configurations described before, and is routinely used to refine structures of average unit cell volumes (between 1000 Å^3^ and 4000 Å^3^), such as those of most ceramic materials, superconductors, etc. The luminosity of the diffractometer is significantly enhanced by the use of curved, focusing monochromators and, thanks to the presence of 32 detectors, the time required to record an entire diffraction pattern, with samples as large as a few grams of material, is reduced to a few hours.

As we have mentioned previously, the observed peak shapes are the convolution of the instrumental profile and the profile generated by the sample. If both these functions are Gaussian with full width at half maximum *H*_1/2_ and *h*_1/2_ respectively, then their convolution is also Gaussian, with width *B*_1/2_ given by

$$B_{1/2}^{2}=H_{1/2}^{2}+h_{1/2}^{2}.$$ (9)

**Fig. 6:**
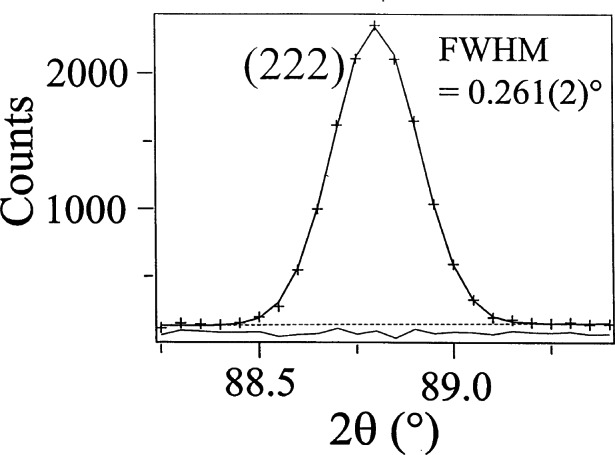
Gaussian fit of the 222 reflection obtained from a powder of the cubic perovskite superconductor MgCNi_3_. The crosses are experimental values and the continuous line the calculated profile, and the difference between the two is shown at the bottom of figure. The good agreement between observed and calculated intensities is proof of the high quality of both the new diffractometer and the sample used in the experiment.

**Fig. 7:**
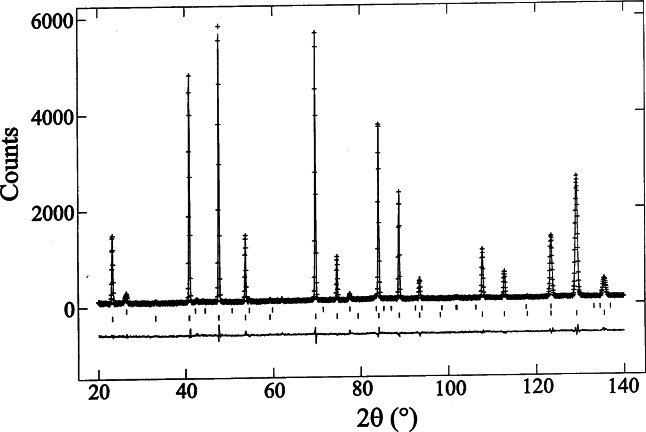
Calculated (continuous line) and observed (crosses) patterns, plotted after the final refinement of the cubic perovskite superconductor MgCNi_3_. The difference between theoretical and experimental values is indicated by the profile in the lower part of figure. The short vertical marks indicate the Bragg positions of the reflections of a small quantity of graphite impurity (high marks) and of the MgCNi_3_ sample (lower marks).

If *h*_1/2_ is small compared to *H*_1/2_ (i.e., the size of the crystallites in the sample is in the range of 1500 Å to 3000 Å with no significant structural distortions and/or strains), then *B*_1/2_ ≈ *H*_1/2_ and Eq. (5) is a valid description of *B*_1/2_ as function of *θ.* If, on the other hand, the pure profile is broadened by crystallite size effects, then we may express *h*_1/2_ by means of Scherrer’s Eq. (8) and introduce it in Eq. (5) obtaining the expression

$$B_{1/2}^{2}=\left[U+\left(K^{2}\lambda^{2}/D^{2}\right)\tan^{2}\theta \right]-V\tan\theta +\left[W+\left(K^{2}\lambda^{2}/D^{2}\right)\right]$$ (10)

where *K* is a constant (≈ 0.9) and *D* is defined as the thickness of the crystallites in the direction perpendicular to the reflecting planes (it should be noted that, *sensu stricto*, Eq. (10) is valid only when the instrumental and pure profiles are both Gaussian). When the crystallites have odd shapes (such as plate-like or needle-like), *D* changes from reflection to reflection and an expression like Eq. (5) cannot be used to describe the dependence of *B*_1/2_ on *θ.* If, on the other hand, the crystallites are approximately spherical and of uniform size, *D* is constant and Eq. (10) has the same form as Eq. (5). It is remarkable that in the majority of cases encountered in practice, this condition is satisfied and the observed peaks are very nearly Gaussian with widths having a dependence on *θ* well described by Eq. (5). Examples of single peaks obtained with the high-resolution diffractometer now in use at the BT-1 beam port are shown in Fig. 6. The excellent quality of the data that can be collected with the instrument is clearly illustrated in Fig. 7, which shows the agreement between observed and calculated intensities after the refinement of the structure of MgCNi_3_ [9]. It is proper here to point out that this unusual and important perovskite compound is a superconductor with *T*_c_ ≈ 9 K, and that its structure was determined and refined by Q. Huang, a guest scientist of the NIST Center for Neutron Research, using a sample prepared in the Department of Chemistry of Princeton University.

### 2.4 Single Crystal Diffractometers

**Fig. 8:**
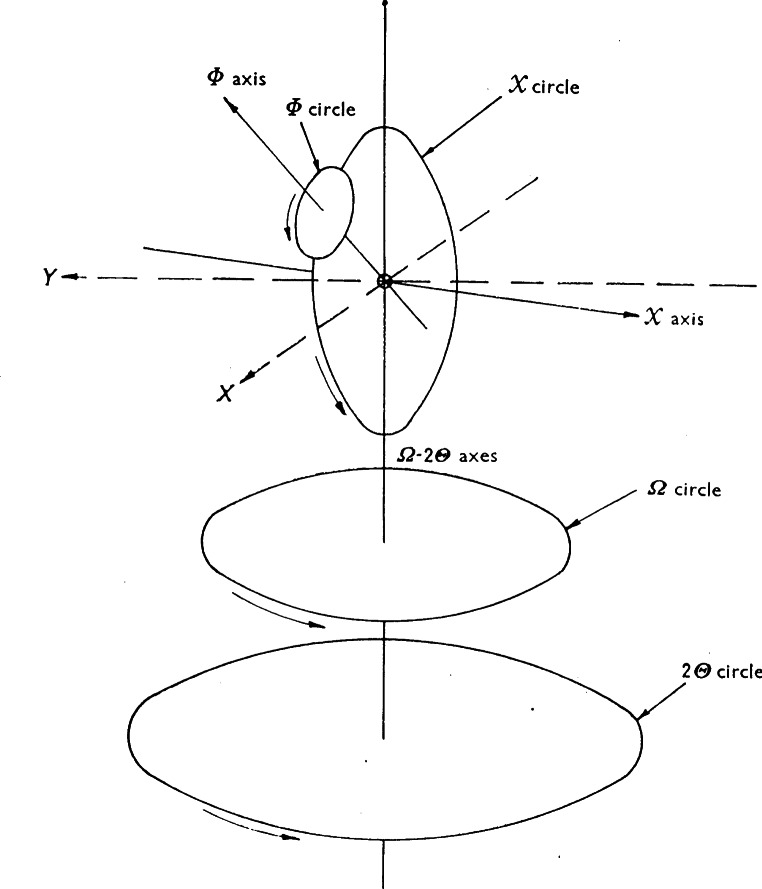
Goniometer head of a four-circle diffractometer used for single crystal diffraction experiments. In this instrument the detector rotates about the 2*Θ* circle axis, and the crystal is located the center of the *χ* circle. Intensities are measured on the horizontal plane, which is parallel to the planes of the *Ω* and 2*Θ* circles. The *ω* rotation (which takes place about the 2*Θ*, but is independent from the *θ*−2*θ* rotation) allows the experimenter to rotate the crystal about the scattering vector of any of the measurable reflections.

Since overlapping of reflections is rarely a problem in single crystal diffraction work, the neutron diffractometers used in this type of analysis are usually designed to maximize luminosity and without paying too much attention to resolution. Consistently with this concept, the single crystal instruments installed at the BT-7 and BT-8 beam ports used graphite or copper monochromators with large mosaic spreads and essentially no collimators. The crystal mounting is in general a three-circle goniometer of the type schematically represented in Fig. 8. The rotations *θ, χ* and *ω* allow the experimenter to bring any reflection *h k l* into diffracting position on the equatorial plane, defined by the incident and diffracted beams, and to rotate the crystal of any angle ψ about the scattering vector. This last option is important in those cases in which multiple diffraction must be avoided [10]. Our original diffractometer was equipped with a single detector rotating about the axis of the 2*θ* circle, and the diffracted intensities were measured following the automatic procedure described in Ref. [11].

**Fig. 9:**
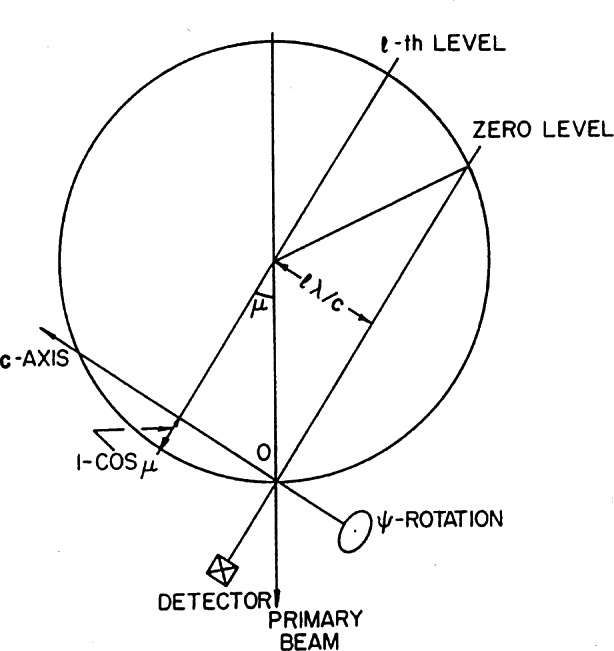
Geometry of the flat-cone Weissenberg method. The *c* -axis (or any other important rational direction of the crystal) is first brought into the plane of figure, at the position indicated, by a goniometer of the type illustrated in Fig. 8. In this position the *l* -th level of the reciprocal lattice, which is perpendicular to the *c* -axis, passes through the center of the reflection sphere. When the crystal is rotated about the *c* -axis by using the appropriate combinations of the *ϕ*, *χ* and *ω* rotations, the reflections of the *l*-th level develop on a vertical plane (also perpendicular to *c*) and can be intercepted by a linear position sensitive detector (whose projection on the plane formed by the primary beam and the *c* -axis is shown in the figure).

During the seventies, the BT-7 beam port was used to develop a new type of single crystal instrument suitable for protein crystallography. Since the unit cells of protein materials are very large, it is essential to design the instrument so that as many reflections as possible are measured simultaneously. Consistently with this requirement, our diffractometer was equipped with a linear position-sensitive detector, and the crystal, mounted on a three-circle goniometer of the type illustrated in Fig. 8, was rotated according to the flat-cone Weissenberg geometry [12]. With this technique, all reflections of any given layer of the reciprocal lattice develop on a single plane, which is made to coincide with the plane formed by the linear detector and the incident beam, as shown schematically in Fig. 9 [13]. This machine became obsolete with the advent of two-dimensional detectors, but in its lifetime valuable data were collected with it, which were used to solve the structure of the bovine pancreatic trypsin inhibitor [14].

## 3. Structural Studies

### 3.1 Neutrons and X-Rays

In order to understand the type of structural problems studied by neutron diffraction at the NBS/NIST reactor is it necessary to briefly summarize the main properties of the neutron radiation and to underline the differences between the neutron techniques and the older and more conventional x-ray methods.

The wavelength of monochromatic beams of thermal neutrons used in structural investigations is of the order of 1 Å, i.e., similar to that of x rays used for the same purpose. The two types of radiation, however, are scattered by atoms in quite different ways. In the case of x rays, the fundamental scattering is generated by the extra nuclear electrons, which, by virtue of their charge, interact with the incident beam. In the case of neutrons, the fundamental scattering is generated by the atomic nuclei, except for magnetic materials where also electronic scattering can be significant (see article entitled “Magnetic Structure Determinations at NBS/NIST” in this Special Issue). The two scattering processes result in qualitative and quantitative differences in the way in which structural experiments are carried out and in the type of results obtained in the two cases, as we will soon describe.

**Fig. 10:**
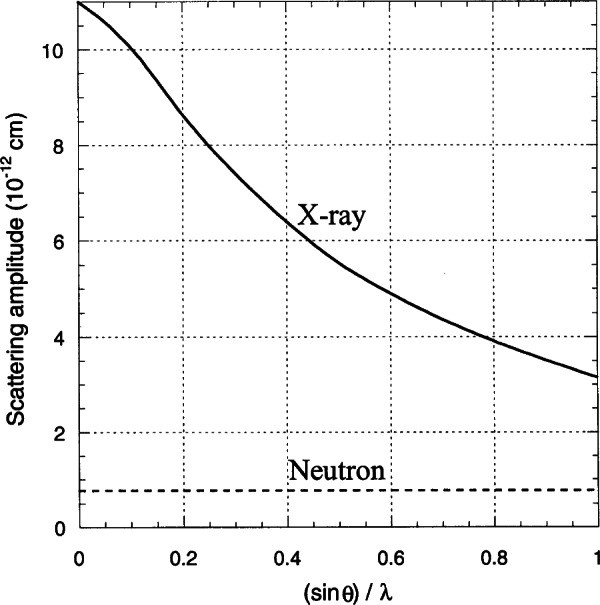
Plot of the neutron and x-ray scattering amplitudes of yttrium versus (sin *θ*)/*λ.*

Since the dimension of the electron clouds surrounding the atomic nuclei are comparable with the wavelengths of the x-rays used in crystallography (0.7 Å to 2.0 Å), the amplitude of the scattered radiation will fall off rapidly when the angle between incident and scattered directions increases. On the other hand, neutron scattering is essentially isotropic because the size of the atomic nuclei is small compared with the wavelength of thermal neutrons. In other words, the scattering amplitude of x rays is function of the diffraction angle, while that of neutrons remains practically constant with *θ.* This behavior is illustrated in Fig. 10, where the x-ray and neutron scattering amplitudes of an yttrium atom are shown as function of (sin *θ*/*λ*). The consequence of this effect is that the intensities of x-ray diffraction patterns are significantly weakened at high values of *θ*, while those of neutrons are not. In practical terms this means that in a neutron experiment we can collect data also at high values of *θ*, where the intensities are very sensitive to both the positional and thermal parameters of the atoms in a structure. It is worthwhile to note that a precise knowledge of the atomic temperature factors is important not only to evaluate appropriate corrections to the bond lengths, but also to understand the role of molecular motions in phase transitions. An example of this application of neutron diffraction is the study by Choi and Prince of several metal azides (specifically, *β*-NaN_3_, KN_3_, RbN_3_, and TlN_3_) done at the NBS reactor [15,16]. In this series of experiments it was shown that the azide ions are linear and centrosymmetric and that their thermal vibrations could be treated as rigid body motions.

As mentioned previously, since the x rays interact with the extra nuclear electrons, the amplitude scattered by an atom is generated by the contribution of all the electrons surrounding the nucleus. It follows that the intensities diffracted by crystals containing heavy and light atoms at the same time will be dominated by the heavy atoms. While this property has been used in the past as a way to solve a large number of crystal structures (especially, but not exclusively, of organic compounds) [17–19], in many cases its presence makes it difficult to locate with precision the light atoms and to determine their thermal and occupancy parameters. In the case of neutrons the scattering amplitude of all atoms is in general of the same order (within a factor of 2 or 3) and does not show a regular dependence on the atomic number. As a consequence, positional and thermal parameters of light atoms can be determined in a neutron experiment even when the number of heavy atoms in a compound is large. Examples illustrating this property of neutrons are numerous in the literature. In the case of the azides mentioned previously, the position of the N atoms could be easily determined in the neutron experiment since the coherent scattering length *b* of N is of the same order as those of the metal atoms (e.g., 0.926 × 10^−12^ and 0.878 × 10^−12^ cm for N and Tl, respectively; note that the atomic numbers of the two elements are 7 and 81). Another example is the 123 superconductor YBa2Cu3O_6+_*_x_* (with 0 ≤*x*< 7), for which the scattering lengths of oxygen, copper, yttrium and barium are 0.581 × 10^−12^, 0.772×10^−12^, 0.775×10^−12^, and 0.507 × 10^−12^ cm, respectively, and the corresponding atomic numbers 8, 29, 39, and 56. This situation has allowed crystallographers to solve the structure with great precision and to determine the number and the location of the oxygen vacancies over the entire range of *x* [20–22].

An additional, important difference between x rays and thermal neutrons is the extent to which they are absorbed in materials. In a significant number of cases, which include the majority of heavy atom elements, the absorption of thermal neutron is negligible, while that of x rays is very large and requires special experimental procedures to reduce its effects and/or the evaluation of complicated corrections to the observed intensities, even when small samples are used. This point is illustrated in Table 1 for the 123 compound YBa_2_Cu_3_O_7_. The absorption of this material for x rays and neutrons of wavelengths 1.54 Å and 1.08 Å, respectively, is expressed in terms of the *mass absorption coefficient (µ/ρ*) (where *µ* is the *linear absorption coefficient* in cm^−1^ and *ρ* density of the sample in gcm^−3^) because this quantity is approximately independent of the physical state of the compound and its value can be obtained, to a good approximation, by adding the mass absorption coefficients of the elements according to the equation

$$\mu /\rho =\Sigma_{i}\gamma_{i}{\left(\mu /\rho \right)}_{i}$$ (11)

where γ*_i_* is the mass fraction contributed by element *i* of mass absorption coefficient (*µ*/*ρ*)*_i_*, and the summation is taken over all the constituent elements *i*. The low value of *µ*/*ρ* evaluated for neutrons means that the attenuation of the primary beam in a neutron experiment is essentially due to the diffraction taking place in the sample (secondary extinction), rather than to *true* absorption. An important consequence of this property is that it makes it possible to use large or bulky samples, thus reducing the data collection time, and to minimize, or eliminate, preferred orientation effects in experiments done with the powder technique. As we will show later, the low absorption of thermal neutron has been used to great advantage in texture studies of bulky samples of metals and alloys.

### 3.2 Crystal Structure Analyses

#### 3.2.1 Solid State Ionics

In general, the structures of ionic conductors are made of two parts, one formed by atoms or ions in fixed positions and the other by ions of high mobility, which account for the ionic conductivity. The atoms in fixed positions form the so-called *framework structure* [24], which may have a wide range of complexity. For example, in *α*-AgI it is a simple body centered arrangement of I ions with cubic symmetry [25]; in Naβ− and β″− alumina it is a block of two or three units of a spinel-like structure [26]; and in some zeolites it may be a still more complex assemblage of cage-like units [27]. An accurate knowledge of the geometry of the framework structure is essential for understanding the conduction mechanisms in these compounds. In fact, the mobility of the disordered ions between sites depends, among other things, on the size of the opening through which the ions move. An example of the analysis of the geometrical factors involved in this process is given in Ref. [28] for the compound Na_4_Zr_2_Si_3_O_12_. In this structure there are theoretically four possible pathways for diffusion, but only one of them is sufficiently large to allow reasonable mobility of the Na^+^ ions at room temperature.

The mobile ions are somehow distributed among sites of multiplicity greater than the actual number of ions in the unit cell, and their diffusion within the framework structure may occur with different mechanisms. In *α*-AgI, for example, the two Ag^+^ cations in the unit cell are distributed over twelve energetically equivalent sites and may move in a three-dimensional system of pathways [25]. In sodium *β″*-alumina, the Na^+^ cations are free to move in two-dimensional layers between the rigid spinel-like blocks [26]. Finally, in materials such as ZrO_2_ stabilized with CaO, the O^2−^ ions are located in well defined crystallographic sites which are only partially occupied, and the conduction takes place through this defective structure, with the oxygen ions diffusing from the occupied to the vacant sites [29].

The above discussion shows that, in order to prepare compounds which are potential ionic conductors it is necessary to start with appropriate framework structures into which the mobile ions are inserted. The insertion reaction changes the chemical and structural nature of the host in ways that are largely unpredictable. A knowledge of the insertion mechanism and of the changes that the inserted ions cause is therefore needed to prepare solid state ionics and/or to predict which framework structures are most favorable for the formation of these useful materials. In particular, compounds which undergo topotactic insertion of lithium are interesting because of their potential use as electrodes in secondary batteries. Lithium is ionic in these materials and the charge introduced by Li^+^ in the host structure is compensated by a corresponding reduction in the oxidation state of the host cations. Since the insertion of extra ions usually causes significant changes which may pulverize the starting materials, it is in general difficult or impossible to grow single crystals of the inserted compounds, and consequently the powder technique, coupled with profile refinement analysis, is the only method for studying this type of structures. As we have discussed previously, neutrons must be used, since they are more sensitive than x-rays in locating the light Li^+^ ions in the structure.

Lithium inserted materials were being studied in the early 1980s at the Ceramics Division of NBS and at Bell Laboratories^1^ in Murray Hill, N. J. It was therefore natural for the Reactor Radiation Division to establish collaborations with these organizations, and a number of problems were studied and solved over the years under this project. In what follows we will briefly discuss, as an example of the work done, the results obtained for the system Li*_x_*ReO_3_.

**Figure 11:**
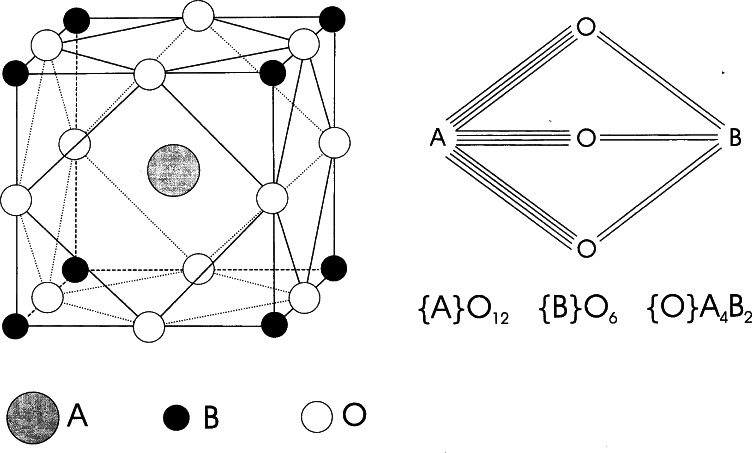
The unit cell of the ideal cubic structure of an ABO_3_ perovskite. The large A cation is at the center of a cuboctahedron and the small B cations at the center of regular octahedra. In order to exist, the A-O and B-O bond distances must be related so that (A-O) = (B-O)(2)^1/2^. On the right side of figure are indicated the bonding scheme and the coordinations of the atoms (atoms in curly brackets are the central, coordinating atoms).

**Fig. 12:**
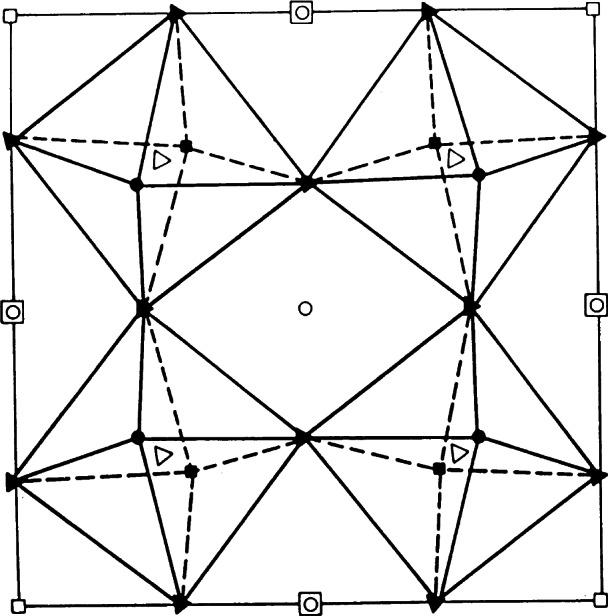
The cubic (*Im*3) structure of Li_0.2_ ReO_3_ projected on the (001) plane. The empty triangles represent Re atoms at *z* = 1/4, 3/4; empty squares: Li at *z* = 1/2; empty circles: Li at 0, 1; full triangles: O at *z*≈1/4, 3/4; full squares: O at *z*≈1/2; full circles: o at *z*≈0, 1. In this structure the ReO_6_ octahedra are tilted of the same angle, and in the same sense, along all three crystallographic axes.

**Fig. 13:**
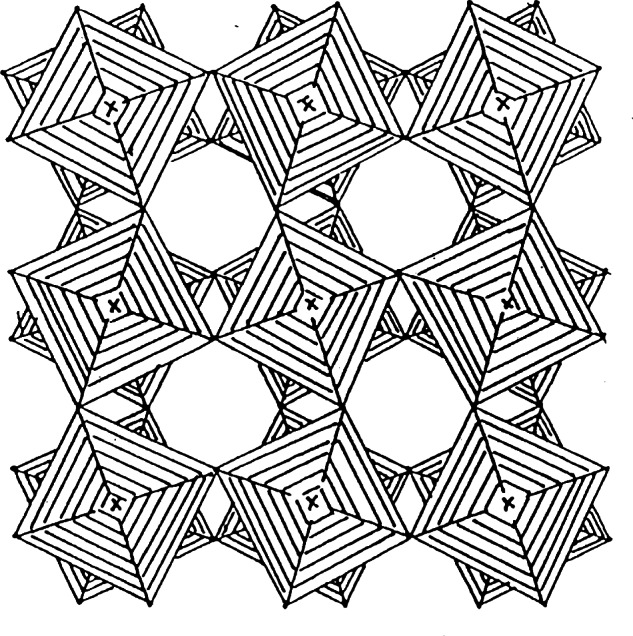
Example of cooperative tilting of the BO_6_ octahedra in the perovskite structure. In this case the octahedra are rotated only about the direction of the vertical axis and in opposite senses in each successive layer. When tilting occurs, the twelve-fold coordination of the large A cations changes drastically, while the BO_6_ octahedra in many cases remain practically undistorted. The oxygen and B atoms are located at the corners and the centers of the octahedra, respectively.

Compounds such as ReO_3_ (and WO_3_) are suitable framework structure for Li^+^ and, in fact the system Li*_x_*ReO_3_ exists in three distinct phases with composition 0 ≤ *x* ≤ 0.35, *x* = 1.0 and 1.8 ≤ *x* ≤ 2.0. The structure of ReO_3_ has the perovskite arrangement of the ReO_6_ octahedra (Fig. 11) and the cuboctahedral cavity, normally occupied by the large A cations in the A B O_3_ perovskites, is in this case empty. For concentrations less than 0.35 Li atoms per formula unit, the Li^+^ ions occupy this cavity [30]. The symmetry for the composition Li_0_._2_ReO_3_ is that of the cubic space group Im_3_, and is produced by a distortion of the original ReO_3_ structure caused by a cooperative rotation of the ReO_6_ octahedra, as illustrated in Fig. 12. The nature of the distortion can be understood if we remember that in the perovskite configuration (see Fig. 11) the octahedra share corners, so that if any one of them is tilted, all the others will also rotate. This situation is illustrated in Fig. 13 for the simple case in which the tilt axis is parallel to the vertical axis of the structure. In Li_0_._2_ReO_3_, the geometry of the final configuration is more complex because a tilt of the same angle occurs about all three crystallographic axes simultaneously. It can be shown [31] that the tilt angle *φ* is related to the *a*-parameter of the *Im*3 structure (*a* = 7.3979 Å) and to the Re-O bond distance (*d* = 1.887 Å) by the equation

cosφ=(3a−4d)/8d (12)

so that, for our structure the tilt angle is *φ* ≈ 14°. It is worthwhile noting that in general, in this type of distortions the octahedra remain essentially the same. For example, the Re-O distances in ReO_3_ and in Li_0.2_ReO_3_ are practically unchanged, the difference being of the order of one hundredth of an Å, and the O-Re-O angles are quite close to 90°.

**Fig. 14:**
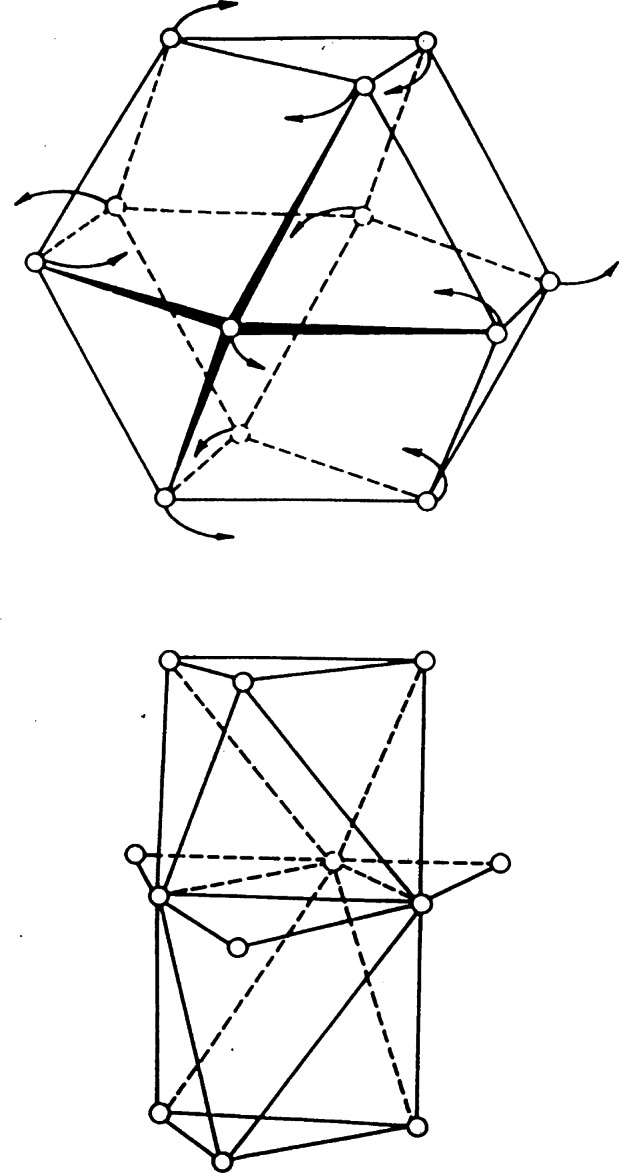
Schematic representation of the distortion of the cuboctahedral cavity (top figure) caused by the tilting of the ReO_6_ octahedra, generating two face-sharing octahedra containing Li atoms in the structures of LiReO_3_ and Li_2_ReO_3_. The oxygen atoms are represented by the empty circles.

The tilt system that accompanies the Li insertion when LiReO_3_ and Li2ReO_3_ are formed is more complex and more extensive than the one discussed previously [32]. In this configuration, the angle *φ* is of the order of 30° and in the resulting structure the cub-octahedral cavity of ReO_3_ is changed into two face-sharing octahedra with six tetrahedra on the re-entrant faces (see Fig. 14). The symmetry of this structure is $R\bar{3}c$ and the two octahedra are fully occupied by Li in Li_2_ReO_3_ and only half occupied in LiReO_3_. This result shows that the tilt system of the ReO_3_ host structure produces a coordination favorable to the insertion of one and two atoms of Li per formula unit, and it is made possible by the fact that the ReO_6_ octahedra share corners.

#### 3.2.2 Crystallography of Superconductors: the System Ba (Pb_1−_*_x_*Bi*_x_*)O_3_

It is important to point out that high-temperature superconductivity in ceramic materials was first observed in the system Ba (Pb_1−_*_x_*Bi*_x_*)O_3_ [33]. Soon after this discovery, structural studies were carried out over the entire range of compositions 0 ≤ *x* < 1 [34–39]. Although the general structural features of Ba(Pb_1−_*_x_*Bi*_x_*)O_3_ do not change dramatically with composition, the type of distortions present in the basic atomic arrangement does vary with *x.* At room temperature, the symmetry changes according to the sequence

**Table:** 

0≤x≤0.05	orthorhombic
0.05≤x≤0.35	tetragonal
0.35≤x≤0.90	orthorhombic
0.90≤x≤1.00	monoclinic

**Fig. 15:**
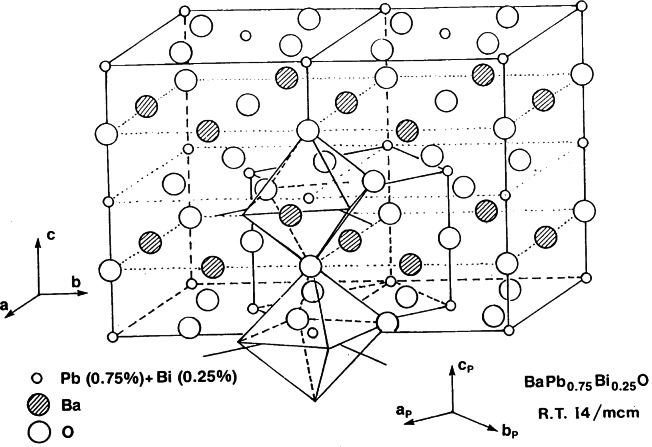
Crystal structure of the compound Ba(Pb_0.75_Bi_0.25_)O_3_. Two unit cells are joined together to show the close relationship with the unit cell of perovskite, defined by the axes *a*_p_, *b*_p_ and *c*_p_. The two octahedra shown in the figure are rotated of the same angle and in opposite senses a bout the *c* -axis, according to the scheme shown in Fig. 13.

Superconductivity exists only in the tetragonal phase. The value of the critical temperature *T*_c_ increases with *x*, reaches a maximum of *T*_c_ ≈ 13 K for *x* ≈ 0.25, and then decreased between 0.25 and 0.35. The arrangement of the atoms in this system is closely related to that of perovskite and the structure of the superconducting tetragonal phase, schematically shown in Fig. 15, can be generated by tilting consecutive (Pb_1−_*_x_*Bi*_x_*)O_6_ octahedra of the same angle (about 8° for *x* = 0.25), but in opposite directions, about the *c*-axis of the unit cell.

For a complete characterization of this system we have to know the oxidation state of the Bi atoms, since two formulations are possible as indicated below

$$\text{Ba}\left({\text{Pb}}_{1-x}^{4+}{\text{Bi}}_{x}^{4+}\right)O_{3}\;\text{and}\;\text{Ba}\left[{\text{Pb}}_{1-x}^{4+}\;\left({\text{Bi}}_{1-x}^{3+}\;{\text{Bi}}_{0.5}^{5+}\right)x\right]O_{3}.$$

Most of the structural studies carried out to determine the valence of Bi have been done with samples of BaBiO_3_ [37,39], in which Bi is located in two crystallographically independent positions of the monoclinic space group I2/*m*. Powder neutron diffraction results have shown that the distances Bi(1)-O and Bi(2)-O are significantly different (2.283 Å and 2.126 Å, respectively) and, from these values, it was concluded that the site Bi(1) is occupied by Bi^3+^ cations, and site Bi(2) by Bi^5+^. X-ray photoemission spectroscopy on single crystal of BaBiO3confirmed that there are two independent positions for Bi [40]. However, the difference in electrons density was found to be too small to justify a complete disproportionation into 3+ and 5+ valence states of Bi. This contradiction was resolved in a joint study at the NBS reactor and at Bell Laboratories. In this research [41] it was shown that the previous result may be explained by assuming a different degree of ordering of the Bi^3+^ and Bi^5+^ cations located at the two inequivalent positions, in the sense that one position is occupied by somewhat more Bi^3+^ than Bi^5+^ and the other position is occupied in the opposite way. These results were corroborated in subsequent neutron diffraction experiments at the reactor [42] which showed that there are in fact two polymorphs of BaBiO_3_, one in which the site Bi(1) has a valence of about 3.5+ and site Bi(2) a valence of 4.5+, and the other in which the two sites have both valence 4.0+, i.e., contain an almost equal proportion of Bi^3+^ and Bi^5+^. The charge ordering between the two sites, i.e., the existence of one polymorph or the other, depends on the method of preparation of the samples and on their thermal history, and in fact, the two phases can be transformed into one another by heat treatment. It is interesting to note that the same assumption of a disordered distribution of the Bi ions has been made to explain the behavior of the system Ba_1−_*_x_* K*_x_* BiO_3_ [43,44].

#### 3.2.3 The System La_2−_*_x_* M*_x_* CuO_4−_*_y_* (M = Ba, Sr)

**Fig. 16:**
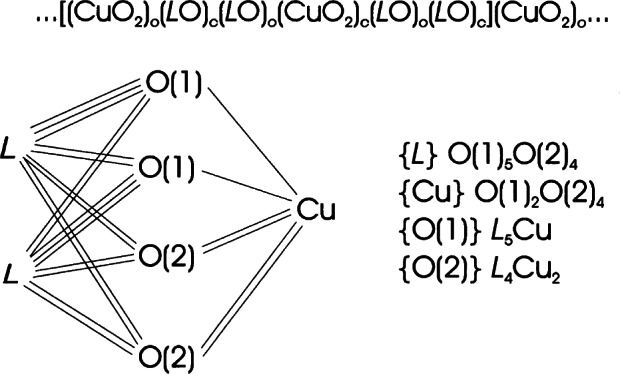
Layer sequence, bonding scheme and atomic coordinations in the structure of (La_2−_*_x_M_x_*) CuO_4−_*_y_* (*M* = Ba, Sr). The symbol L represents the mixed cations La_1−δ_*M*_δ_, Where δ = *x*/2. In the sequence, the formulas enclosed in the parentheses give the chemical composition of each layer and the subscripts o and c indicate if the cations are at the origin or in the center, respectively, of the mesh characteristic of the layer. The square brackets include the content of one unit cell of the structure.

The discovery of superconductivity in this system was made on a sample consisting of a mixture of phases [45], and only in subsequent experiments was the superconducting material identified as La_2_-*x*Ba*_x_*CuO_4−_*_y_* [46,47]. This compound has the K_2_NiF_4_-type structure at room temperature, consisting of alternate layers with the perovskite and rocksalt structure, as illustrated in Fig. 16. The value of the critical temperature *T*_c_ is also, in this case, a function of *x* and reaches its maximum of about 35 K for *x* ≈ 0.15. The detailed structure for this composition was determined by neutron powder diffraction [48,49] which showed that the symmetry at room temperature is tetragonal *I*4/*mmm* and that a transition to an orthorhombic phase with space group *Cmca* occurs at about 180 K [49]. The lattice parameters of the two phases are related so that *a*_0_ ≈ *c*_0_ ≈ *a*_t_ $\sqrt{2}$ and *b*_0_ ≈ *c*_t_, where *a*_0_, *b*_0_, and *c*_0_ are the parameters of the orthorhombic cell and *a*_t_ and *c*_t_ these of the tetragonal cell.

**Fig. 17:**
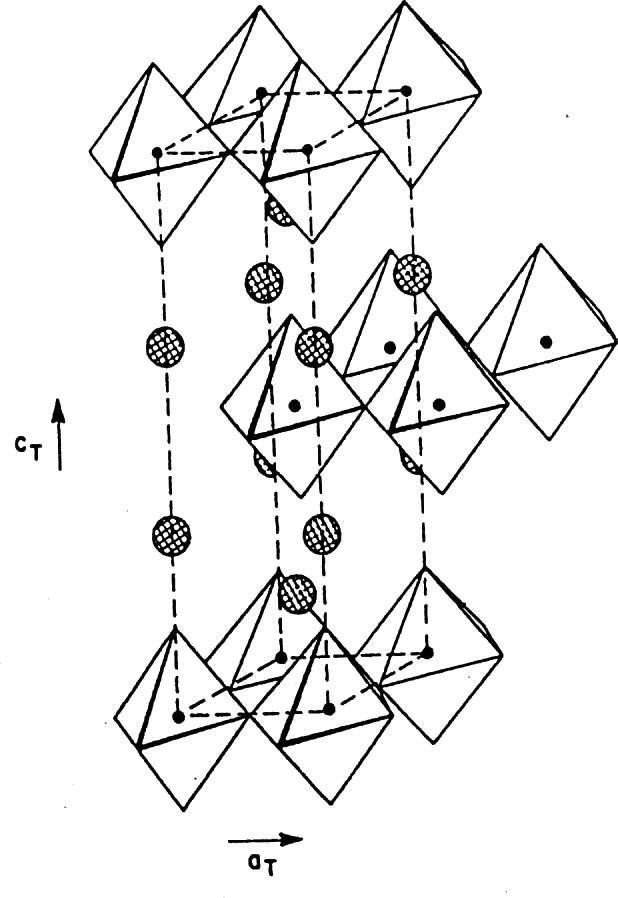
View of the room temperature tetragonal structure of La_1.35_Sr_0.15_CuO_4_. The large shaded circles indicate the mixed cations La_0.925_Sr_0.075_, the small filled circles represent copper atoms at the center of bi-pyramids and the oxygen atoms are located at the corners of the polyhedra.

**Fig. 18:**
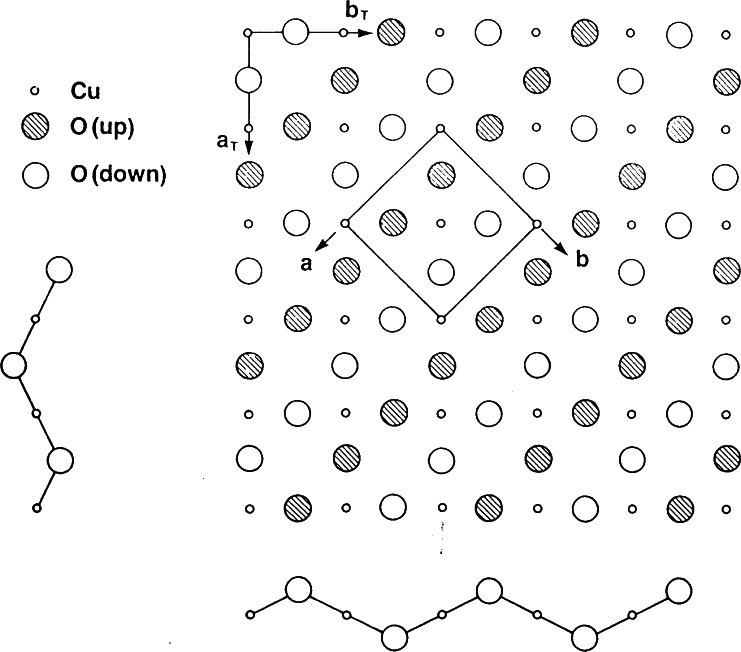
Schematic representation of the distortion of the copper-oxygen layers in the orthorhombic phase of La_1.85_Sr_0.15_CuO_4_. The central part of the figure is the projection of one CuO_2_ layer on the plane of the Cu atoms along the *c*-axis, and the figures at the bottom and the left sides are projections along *a*_r_ and *b*_r_, respectively.

At this stage attempts were being made to prepare compounds with values of *T*_c_ higher than 35 K, and the system La_2−_*_x_*Ba*_x_*CuO_4−_*_y_* was considered as a candidate at Bell Laboratories. The composition *x*≈0.15 was found to have *T*_c_ ≈ 40 K and its structure was refined at the NBS reactor using neutron powder diffraction data [50]. This analysis showed that the Ba and Sr compounds are isomorphic at room temperature and that also the Sr compound undergoes a phase transition from tetragonal to orthorhombic at about 200 K. At all temperatures, the lanthanum and strontium atoms were found to be distributed at random over the same equivalent positions. A schematic picture of the tetragonal structure is shown in Fig. 17, where the perovskite-like planes are emphasized to illustrate the two-dimensional nature of the copper-oxygen layers perpendicular to the *c*-axis. These layers are separated by the La/Sr-oxygen planes, so that the copper atoms in one plane do not share oxygen atoms with copper atoms located on other planes. The Cu-O distances within the perovskite-like planes are short (about 1.89 Å) while those in the perpendicular direction are long (about 2.41 Å), and consequently the coordination polyhedron around copper is a bi-pyramid, rather than an octohedron. The shape of the bi-pyramid changes in a subtle way when the symmetry is lowered from tetragonal to orthorhombic. More specifically, the base becomes rectangular and the copper-oxygen planes are buckled as a consequence of small but significant rotations of the bi-pyramids about axes parallel to the a- and b- directions, as shown in Fig. 18. The neutron diffraction studies of the Ba and Sr compounds showed no oxygen deficiency. This means that the charge compensation for the substitution of La^3+^ with Ba^2+^ or Sr^2+^ must be accomplished entirely by oxidation of copper from 2+ to 3+. By assuming a simple ionic model, the oxidation state of copper in La_1_._85_*M*_0.15_CuO_4_ is 2.15+, i.e., the Cu sites are occupied by Cu^2+^ and Cu^3+^ in the ratio 17/3.

#### 3.2.4 The System YBa_2_Cu_3_O*_x_*

**Fig. 19:**
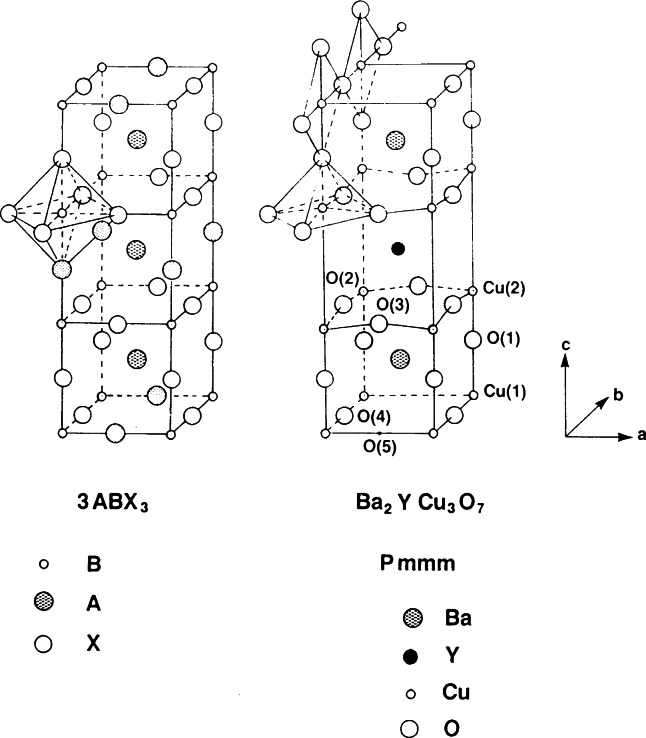
View of the structure of the 94 K superconductor Ba_2_YCu_3_O_7_ (right side) and of the configuration produced by stacking along *c* three unit cells of the ideal structure of perovskite ABO_3_ (left side). The atomic arrangement of the superconductor differs from that of perovskite because (i) the Y and Ba atoms are ordered; (ii) the Y layers and the sites O(5) are not occupied by oxygen atoms; (iii) the CuO_2_ layers are buckled. In Ba_2_YCu_3_O_7_, the Cu(l) atoms are in four-fold planar coordination, resulting in the characteristic chains which propagate along the b-axis of orthorhombic unit cell.

**Fig. 20:**
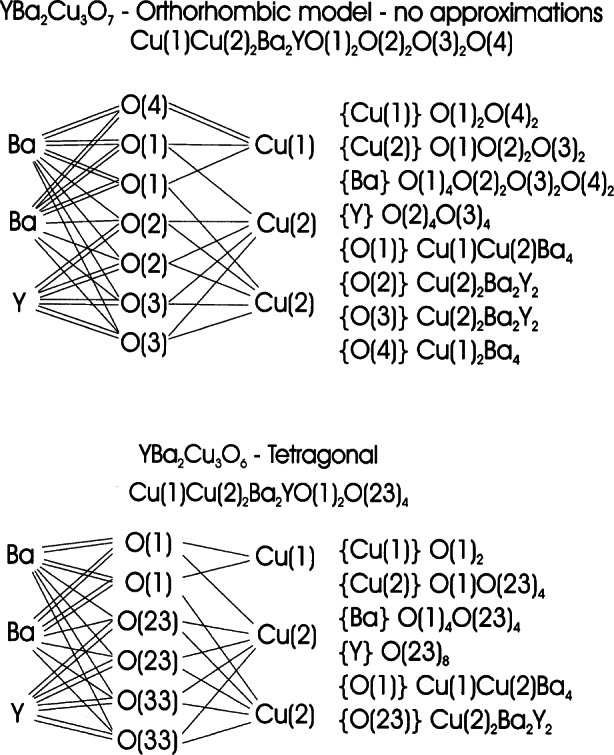
Formula units, bonding schemes and atomic coordinations in the structures of orthorhombic and tetragonal Ba_2_YCu_3_O_7_ and Ba_2_YCu_3_O_6_.

Superconductivity with *T*_c_≈94 K was discovered in a sample which was a mixture of phases [51] and, again, the superconducting compound was identified later in several laboratories as YBa_2_Cu_3_O*_x_*, with *x*≈7.0 [52–54]. Profile analysis based on neutron powder data quickly gave an accurate description of the structure which is shown in Fig. 19, next to the structure of perovskite to underline the similarities and the differences between the two atomic arrangements. The refined structural parameters obtained in some of the most accurate neutron diffraction studies of the composition *x*≈7.0 are given in Table 2, which has been taken from Ref. [55], and the bonding scheme and the atomic coordinations are illustrated in Fig. 20.

Figure 19 shows that the structure of YBa_2_Cu_3_O_7_ is obtained from that of perovskite by tripling the *c*-axis, by eliminating in an orderly fashion some of the oxygen atoms, and by ordering the Y and Ba atoms along the *c*-axis of the unit cell. The copper atoms are located on two crystallographically independent sites of the orthorhombic space group *Pmmm*. The first, labeled Cu(1), has four-fold planar coordination in which the near square CuO_2_ units share corners and form chains along the *b*-axis, and the second, Cu(2) has a pyramidal coordination with two Cu(2)-O(2) and two Cu(2)-O(3) short bonds (1.930 Å and 1.961 Å, respectively) and one Cu(2)-O(1) long bond (2.295 Å). Because of this peculiar structural feature, there are in the structure two-dimensional layers of composition CuO_2_ perpendicular to the c-axis. The oxygen atoms in the layers are slightly shifted from their ideal perovskite positions, producing a buckling of the Cu(2)-O(23) bonds, as indicated in Fig. 19.

It was soon observed that the oxygen stoichiometry in YBa_2_Cu_3_O*_x_* may be lower than seven atoms per formula unit, depending on the preparation and the thermal history of the sample, and that the oxygen vacancies associated with the decrease of the oxygen content involve only the O(4) sites of the CuO_2_ chains [20]. When the number of vacancies becomes sufficiently large, the sites O(5), which are empty for *x* = 7.0, become gradually filled and, at some point, the structure from or thorhombic *Pmmm* becomes tetragonal *P*4/*mmm.* The transition is a function of temperature and oxygen content, and, using appropriate procedures, the orthorhombic modification can be retained at compositions as low as *x* = 6.3. The value of the critical temperature *T*_c_ decreases as *x* decreases, and it becomes equal to zero when the orthorhombic to tetragonal transition takes place. When *x* = 6.0, all O(4) and O(5) sites are empty, and the coordinations of Cu(1) and Ba became two and eight-fold, respectively [58].

The structural changes occurring in YBa_2_Cu_3_O*_x_* over the entire range of compositions 6.0≤*x*<7.0 have been studied in two accurate and extensive sets of neutron diffraction experiments [21,22], and the interpretation of these observations has been provided by Brown, using the bond valence method of analysis [59,60]. By comparing the *a*-axis parameter measured experimentally with that calculated from the theoretical Ba-O(1), Cu(1)-O(4) and Cu(2)-O(23) distances consistent with different valences of the chain and pyramidal copper atom, Brown was able to prove that the oxidation states of Cu(1) and Cu(2) in YBa_2_Cu_3_O_6_ are 1+ and 2+ respectively, while they are approximately the same (2.33 +) for *x* = 7.0. This means that adding oxygen to the O(4) sites on the plane of the Cu(1) atoms increases not only the oxidation state of Cu(1), but also that of Cu(2), i.e., a charge transfer between the two copper atoms takes place during the oxidation, or reduction, of the compound. The Ba-O (1) and Cu(2)–O(23) bonds at *x* = 6.0 are strained because they are incommensurate when the oxidation states of Cu(1) and Cu(2) are 1+ and 2+. As the oxidation progresses, however, the incommensurability of these bonds decreases, and the strains are almost completely relieved at *x* = 7.0. This behavior, therefore, suggests that the observed charge distribution (and all the properties related to it) at different oxygen stoichiometries is a consequence of the commensurability of the Cu-O and Ba-O bonds.

#### 3.2.5 The System RBa_2_Fe_3_O_8+_*_x_*

The substitution of copper by other metal species in the structure of YBa_2_Cu_3_O*_x_* has been used extensively to investigate the correlation between the changes induced into the structure by the substituting cations and the variation of the superconducting properties. The first studies in this direction involved the partial replacement of either the chain copper atoms Cu(1), e.g., [61], or the plane copper atoms Cu(2), e.g., [62]. From these experiments it was found that the introduction of dopants decreases the value of Tc and that the change is very drastic when the atoms Cu(2) are replaced even by small amounts of other elements such as Zn and Ni. These results were taken as evidence that the two-dimensional Cu(2)O(23)_2_ layers play a fundamental role in the superconducting process. Full replacement of copper by other elements, therefore, is important for understanding why copper, of all transition metals, seems to be necessary for superconductivity to occur in this structural type. For this reason collaboration between the NBS reactor and the University of Oslo was established in the early nineties to study the system in which copper is entirely replaced by iron.

**Fig. 21:**
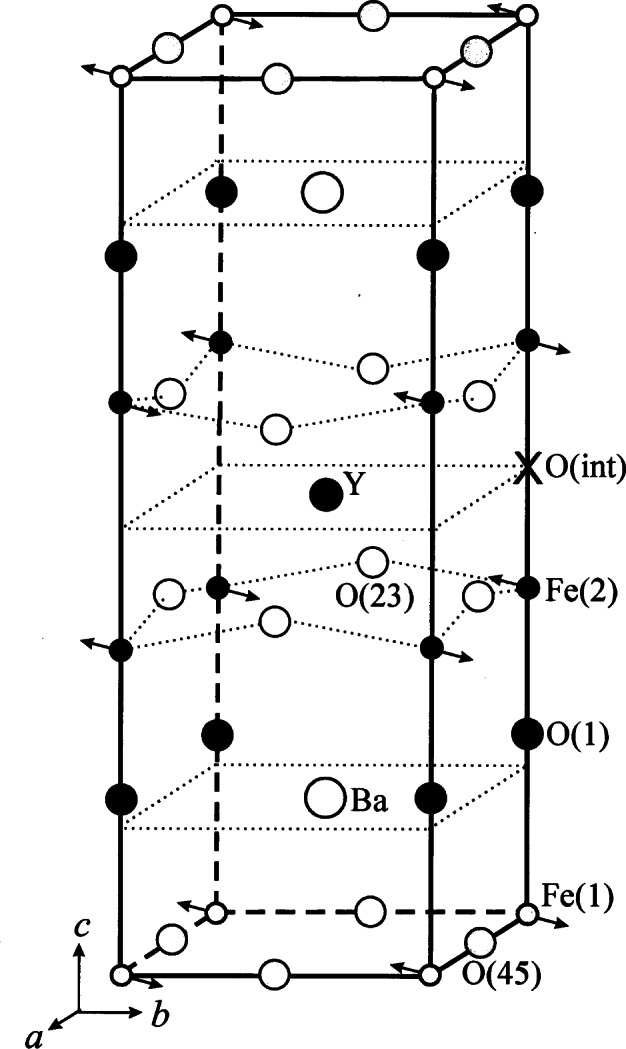
View of the tetragonal crystal structure of YBa_2_Fe_3_O_8_. To facilitate comparison, the labeling of the atoms is consistent with that of Fig. 19. The directions of the magnetic moments of the two crystallographically independent iron atoms are indicated by arrows.

The first compound investigated in this series of experiments was stoichiometric YBa_2_Fe_3_O_8_ [63]. The nuclear structure of this material has the symmetry of space group *P*4/*mmn* and is illustrated in Fig. 21. The configuration of the atoms in the unit cell is very similar to that of the superconductor YBa_2_Fe_3_O_7_, with the exception that the iron atoms Fe(1), corresponding to Cu(1), have octahedral coordination, rather than fourfold planar, and the Fe(1)O_6_ octahedra are arranged in layers. This significant difference is a consequence of the fact that all possible oxygen sites on the Fe(1) plane are fully occupied, giving an oxygen content of eight atoms per formula unit. A second consequence is that the Ba atoms have twelve-fold cuboctahedral coordination. This configuration results in a rather strained structure in which the polyhedron around Ba is considerably compressed.

During the structural refinements based on neutron diffraction data, it was realized that the nuclear structure of the iron compound did not account for all the observed intensities, and especially for those of some strong reflections at low values of the 2 *θ* angle. Polarized neutron diffraction experiments revealed that these intensities have magnetic origin, and a subsequent analysis showed that they are consistent with a magnetic structure in which the iron moments lie on the *a, b* plane and are coupled antiferromagnetically within each FeO_2_ layer, as well as along the *c*-axis, as indicated by the arrows associated with the Fe atoms in Fig. 21. The magnetic moments of the two iron atoms were found to be equal to each other (3.49 *µ*_B_ in both cases) thus showing that the oxidation states of Fe(1) and Fe(2) are also the same (3+). The results of our study were confirmed later by x-ray powder diffraction, magnetic susceptibility and Mössbauer spectroscopy [64, 65]. In another experiment, however, the differences between the magnetic hyperfine fields at Fe(1) and Fe(2) were interpreted as evidence that the valence states and the magnetic moments of the two iron sites are not the same [66]. In order to resolve this uncertainty we have carried out a complete bond-valence analysis of the structure and found that the atomic configuration of the iron compound is consistent with an equal distribution of charge on the Fe(1) and Fe(2) sites, thus confirming our original conclusion based on the determination of the magnetic structure [67].

Soon after the original study described in Ref. [63], it was realized that YBa_2_Fe_3_O_8+_*_x_* can exit in a narrow range of compositions, with −0.2 <*x*< 0.1. The location of the oxygen in excess of the O_8_ stoichiometry was determined in the analysis of the Ca-doped compound (Y_1−_*_y_* Ca*_y_*) YBa_2_Fe_3_O_8+_*_x_* [68]. As indicated in Fig. 21 these atoms occupy the O(int.) sites, on the plane of the Y atoms. Their presence in the structure introduces significant disordering of the atoms in the O(23) sites and increases the average coordination of Y and Fe(2). On the other hand, the presence of oxygen vacancies involves the O(45) sites and cause a decrease of the average coordination of Ba and Fe(2).

#### 3.2.6 Hg-Based Superconductors

**Fig. 22:**
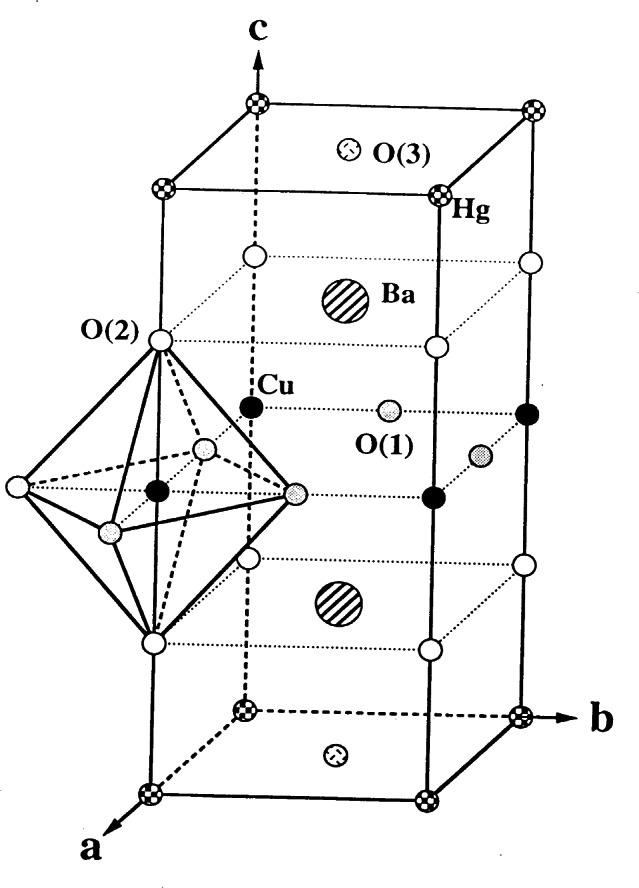
Unit cell of the crystal structure of the 94 K superconductor Hg Ba_2_CuO_4+_*_x_.* In this cell, oxygen sites O(3) are only partially occupied. The copper atoms are in a bi-pyramidal coordination similar to that of Fig. 17. The Ba atoms are in eight-fold coordination in those unit cells in which O(3) is empty and in nine-fold coordination in the others. Similarly, Hg may have two-, three-, four-, five-, and six-fold coordination, depending on how the O(3) sites occupied by oxygen are clustered together.

In the early nineties, superconductivity with *T*_c_ ≈ 94 K was discovered in the mercury based compound HgBa_2_CuO_4+_*_x_* [69]. The structure of this material was first determined by x-ray powder diffraction and is schematically illustrated in Fig. 22. The atomic configuration of this compound can be conveniently described by means of the layer sequence

$$\ldots \left[{\left(\text{BaO}\right)}_{c}{\left({\text{HgO}}_{x}\right)}_{0}{\left(\text{BaO}\right)}_{c}{\left({\text{CuO}}_{2}\right)}_{0}\right]{\left(\text{BaO}\right)}_{c}\ldots$$

where the square brackets include the content of one unit cell and the subscripts c and o indicate if the cation is at the center or at the origin, respectively, of the mesh of each layer. The formulae in parentheses indicate the chemical composition of the layers. Since the extra oxygen atoms O(3) located on the plane of the Hg atoms are necessary to increase the oxidation state of copper, it is important to know how many of them can be incorporated in the structure and where exactly they are located on the layer. As we have mentioned earlier, x rays are not sufficiently sensitive to small amounts of oxygen in the presence of heavy atoms such as Ba and Hg, and for this reason the compound was studied at the NIST reactor in a collaborative project with the French and Russian authors who discovered this important material [70].

The copper atoms in this structure (see Fig. 22) have a bi-pyramidal coordination similar to that found for La_1.85_Sr_0.15_CuO_4_, with an apical Cu-O(2) distance of 2.78 Å, compared with the in-plane distance Cu-O(1) of 1.94 Å. Most of the Ba atoms have eight-fold coordination. However, those located below and above the atoms O(3) are nine-coordinated and the coordination polyhedron may be considered a mono-capped square antiprism. Similarly, the Hg atoms are generally two-coordinated, except in those unit cells in which the sites O(3) are occupied by oxygen atoms.

**Fig. 23:**
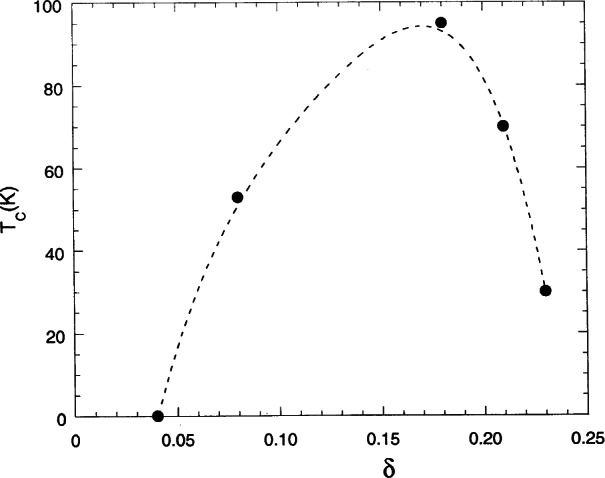
Plot of *T*_c_ versus the composition x for the superconductor Hg Ba_2_CuO_4+_*_x_*.

The compound HgBa_2_CuO_4+_*_x_* is the first member of a homologous series of formula HgBa_2_R*_n_*_−1_Cu*_n_* O_2_*_n_*_+2+_*_x_* and having a structure represented by the layer sequence

$$\ldots \left[{\left(\text{BaO}\right)}_{c}{\left({\text{HgO}}_{x}\right)}_{0}{\left(\text{BaO}\right)}_{c}{\left({\text{CuO}}_{2}\right)}_{0}\left\{\left(n-1\right){\left(R\right)}_{c}{\left({\text{CuO}}_{2}\right)}_{0}\right\}\right]{\left(\text{BaO}\right)}_{c}\ldots$$

where R is an element situated on a layer that does not contain oxygen atoms and is therefore similar to the yttrium layer in YBa_2_RCu_3_O*_x_*. Thus, the layer sequence of the compound HgBa_2_Cu_2_O_6−_*_x_*, corresponding to *n* = 2 is

$$\ldots \left[{\left(\text{BaO}\right)}_{c}{\left({\text{HgO}}_{x}\right)}_{0}{\left(\text{BaO}\right)}_{c}{\left({\text{CuO}}_{2}\right)}_{0}{\left(R\right)}_{c}{\left({\text{CuO}}_{2}\right)}_{0}\right]{\left(\text{BaO}\right)}_{c}\ldots$$

and so on. The sequences show that the structures of these compounds are made by blocks … (CuO_2_)_0_(R)_c_(CuO_2_)_0_ … having the configuration of perovskite, and by blocks … (BaO)_c_(HgO*_x_*)_0_(BaO)_c_… with the rock-salt structure. To avoid writing complex formulae, these materials are usually identified by indicating the number of the Hg, Ba, R, and Cu cations, respectively. Thus, the first member of the series is 1201, the second 1212, etc.. All members have the tetragonal symmetry of space group *P*4/*mmm* and approximate lattice parameters *a* ≈ 3.85 Å and *c* ≈ 9.5 + 3.2 (*n*−1) Å. The oxygen content *x* depends on the preparation method and the annealing conditions of the sample. The maximum value of *x* in each member of the series, however, seems also to be function of *n*, i.e., of the number of layers that constitute the perovskite block [71, 72]. The critical temperature *T*_c_ is strongly dependent on *x*. An example of the behavior of *T*_c_ is provided by the 1201 compound and is illustrated by the graph of Fig. 23, taken from Ref. [73].

For *x* < 0.06, *T*_c_ is equal to zero, then it increases with increasing *x*, reaches a maximum of *T*_c_ ≈ 94 K for *x* ≈ 0.17, and then decreases. Similar behavior has been observed also for other members of the series. For each of these compounds, in which R = Ca, the maximum value of *T*_c_ is a function of *n*, and, more specifically, it increases for increasing *n* up to *n* = 3, and then decreases as shown Table 3.

## 4. Zeolitic Materials

(This section was written by B. H. Toby, Leader, Crystallography Team)

### 4.1 Introduction

Zeolites and related microporous materials (herein referred to as zeolitic materials) are crystalline materials consisting of alternating regions of framework atoms and voids. The framework is typically composed of tetrahedral aluminum and silicon atoms linked by oxygen atoms and the voids or pores can range to just accommodating a water molecule, to pores large enough to fit a C_60_ molecule or DNA fragment.

Zeolitic materials frequently form highly symmetric and complex structures that are elegant to behold. Further, these materials are industrially important for a number of diverse applications. At least one zeolite-based catalyst, and likely more than one, is employed to produce all domestic gasoline. Zeolitic catalysts are in creasingly used for the preparation of pharmaceuticals and other fine chemicals. Zeolitic materials have novel capabilities, which can often be enhanced to perform a particular chemical separation. They are frequently used commercially to separate gases, for example producing N_2_ or O_2_ from air. Another application is to absorb water, for example to keep double-pane windows from fogging.

Most zeolitic materials have negatively charged frameworks that require cations for charge balance. This allows these materials to be used for ion exchange. In our homes, zeolites are formulated in detergents to remove the calcium ions that make water hard. Different materials are used to sequester radioactive ions for environmental remediation.

Zeolitic materials rarely crystallize as single crystals. This means that most structural studies must utilize powder diffraction. Further, these materials frequently contain both light and heavy atoms and thus neutron scattering information is essential to determine sitting of light atoms. The use of both neutron and synchrotron x-ray diffraction data in many cases has been a prerequisite for accurate structural determinations (for example, see [74]).

The following paragraphs summarize results from many recent studies performed at NIST where neutron diffraction played a pivotal role.

The 32 detector BT-1 neutron powder diffractometer in the NIST Center for Neutron Research allows the instrumental resolution to be tailored to suit the needs of the sample[75]. This has been very effective for the study of zeolitic materials, since optimum resolution is needed at relatively low *Q* values, as opposed to dense-phase materials. The instrument was further improved for zeolitic materials in the mid-1990s, when the 1.54 Å Cu(220) 75° takeoff angle monochromator was replaced with a Ge(311) monochromator with a wavelength of 2.078 Å. Data can be collected approximately twice as fast with Ge(311) compared with the Cu(220) monochromator and resolution is even better tailored to the needs of zeolitic diffraction experiments.

### 4.2 Zeolite RHO

**Fig. 24:**
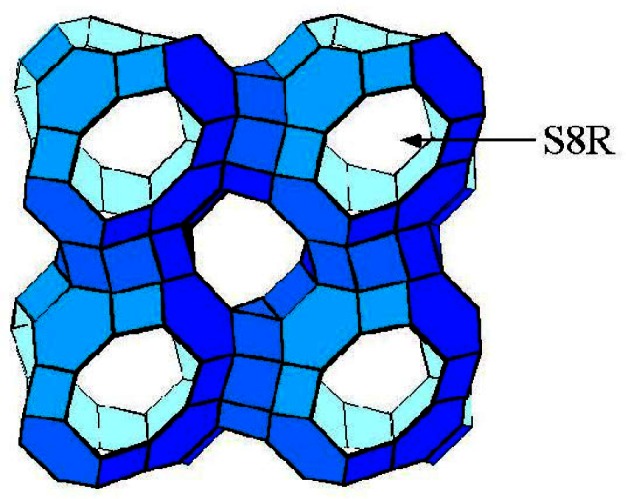
Schematic representation of the Zeolite RHO framework. Vertices indicate tetrahedral atoms (oxygen atoms are omitted for clarity). The single eight-ring (S&R) pore opening is indicated.

**Fig. 25:**
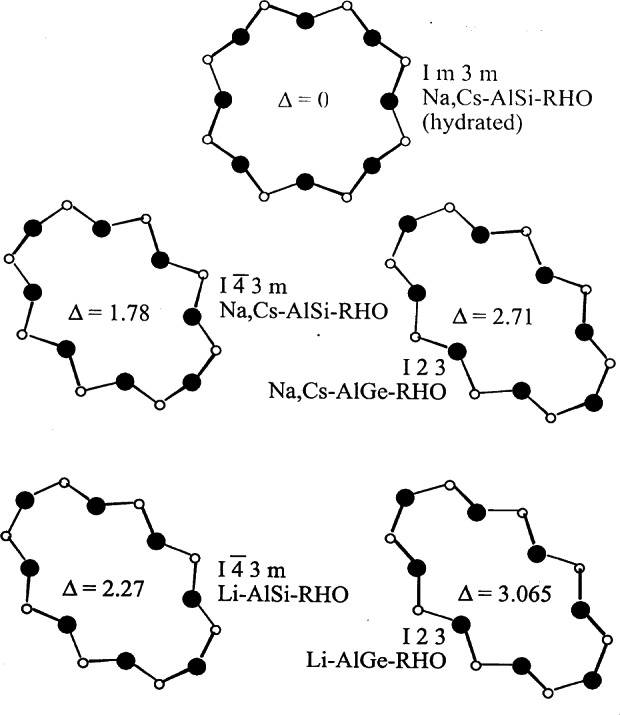
Configuration of the Single Eight Ring in Zeolite RHO materials. Tetrahedral atoms are shown as open circles; oxygen atoms are shown as larger filled circles. The Δ parameter indicates the degree of distortion.

The zeolite RHO framework is shown in schematically in Fig. 24. This material has been of interest in part due to the extreme flexibility of the framework, as shown in Fig. 25. Cesium-exchanged zeolite RHO was one of the first materials to be studied in the early 1970s on the then new five-detector BT-1 instrument [76]. The framework topology has continued to be of interest. In one recent study, an aluminosilicate RHO material, Li_7.6_Cs_1.3_Na_2.0_Al_11.4_Si_36.6_O_96_, was compared to a novel aluminogermanate RHO material, Li_13.9_Cs_5.24_Na_0.24_Al_24_Ge_24_O_96_ [77]. This study demonstrated that despite having the same framework, the different compositions resulted in different cation sites. In these systems, Li sites could not be determined without use of neutron diffraction.

The high degree of flexibility in the RHO framework leads to what is known as the trap door effect with some cations, such as Cd. At room temperature, the cation binds in the single-eight ring window (S8R, see in [24]) but at elevated temperatures, the cations migrate to higher coordination sites. Initial reports indicated that when the material was cooled, the cations returned to the S8R sites [78, 79]. When the cations are located in the S8R site, they block access to the zeolite pores. Thus it is possible to trap species in this material, by sorbing the guest at elevated temperatures. When the temperature is lowered, the guest is trapped when the cations return to the S8R sites. Subsequent study indicated that cations are stable in the S8R site only when hydrated and that H_2_O (or possibly OH−) remains bound to the cations at much higher temperatures that previously understood [80]. With heating, the water desorbs and the cation migrates to a higher coordination site. The cation remains in this higher coordination site when the temperature is reduced, except in the presence of water vapor. Thus, the trap door effect is actually a hydration/dehydration phenomenon rather than simply a temperature-mediated effect. These results also call into question the observation of negative thermal expansion in Sr exchanged RHO [81, 82]. It is now believed the contraction of the unit cell upon heating is due to a reversible loss of water.

### 4.3 Structure-Directing Agents in Synthesis of CON Framework Materials

**Fig. 26:**
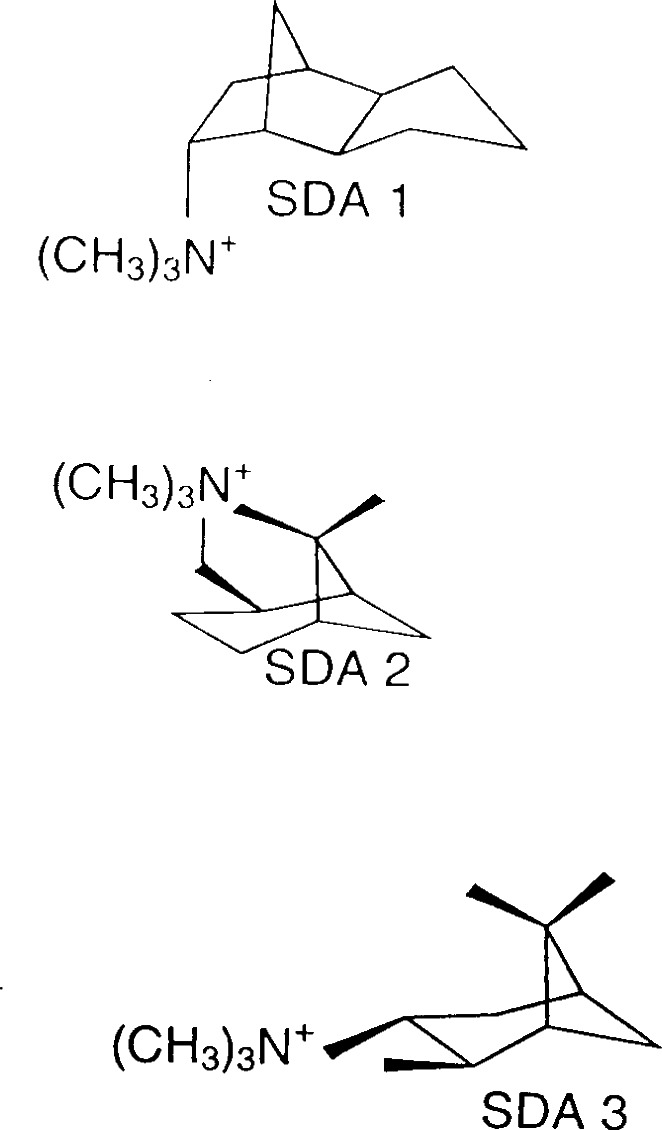
Structure-directing agents studied for preparation of CON framework materials.

Many zeolitic materials are prepared from gels containing a sacrificial organic amine, called a structure-directing agent (SDA), that is trapped in the pores during synthesis. To free the pore spaces, the SDA is oxidized away by calcinations in air. The influence of the SDA species during crystallization is poorly understood. Indeed a single SDA can be used to produce different materials from the same gel composition, by variation of processing conditions [83]. A study combining neutron and synchrotron diffraction with molecular mechanics was recently undertaken to investigate how SDA cations interact with a set of zeolitic materials [84]. The borosilicate zeolitic materials, SSZ-33 and CIT-1 have related structures and have been designated the structure code CON by the Structure Commission of the International Zeolite Association. Unlike CIT-1, which is nearly free of defects, SSZ-33 has a very high density of stacking faults (≥30 %) [85]. Several organic cations, such as species SDA 1 shown in Fig. 26, can be used to synthesize SSZ-33. However, only SDA 2 has been found to synthesize CIT-1. Interestingly, cation SDA 3, while quite similar to SDA 2, cannot be used to prepare any material in the CON family.

**Fig. 27:**
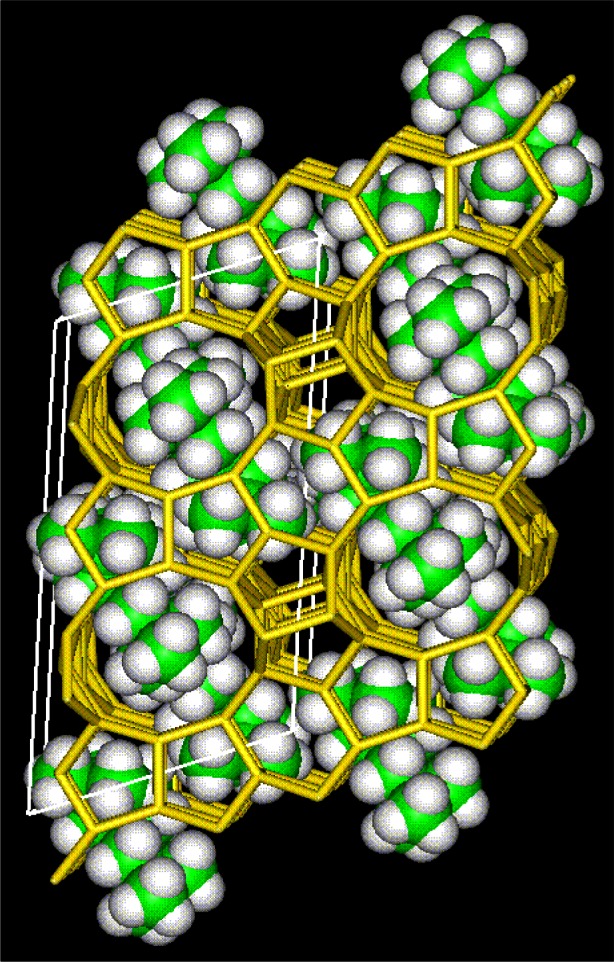
Packing of SDA-2 in CIT-1. Carbon atoms are shaded and hydrogen atoms are shown in white, both at Van der Waals radii; nitrogen atoms cannot be seen. The CIT-1 framework is shown as dark lines, where oxygen atoms have been omitted.

The location of the SDA cation in CIT-1 prior to calcinations was determined using a simultaneous Rietveld fit to both neutron powder diffraction data and synchrotron x-ray powder diffraction data. Determination of the cation orientation was made possible by selective deuteration of the methyl groups of the quaternary nitrogen. Since crystallographic methods do not prove uniqueness of the resulting model, molecular modeling was used to tabulate all possible sites where the SDA cation could be accommodated in the CIT-1 pores. Based on these computations, it was concluded that the crystallographic model is the only plausible result. This model, shown in Fig. 27, demonstrates the very tight fit between the SDA ions and with the CIT-1 framework.

Subsequent molecular modeling work was performed using the crystallographic model as a starting point. These results confirmed that four molecules of either SDA 1 or SDA 3 can pack in the CIT-1 pores without any unfavorable energetic interactions and with similar energetic. However, packing SDA 3 in the same voids required significantly more energy, due to Van der Waals repulsions. This suggests why SDA 3 does not synthesize the CON framework, since it cannot pack effectively to form the same voids. Molecular modeling of SDA packing in the presence of stacking faults showed no significant energetic differences between SDA 1 and SDA 2, indicating that these stacking faults likely arise as a kinetic effect rather than due to thermodynamics.

### 4.4 Novel Lithosilicate Molecular Sieves

**Fig. 28:**
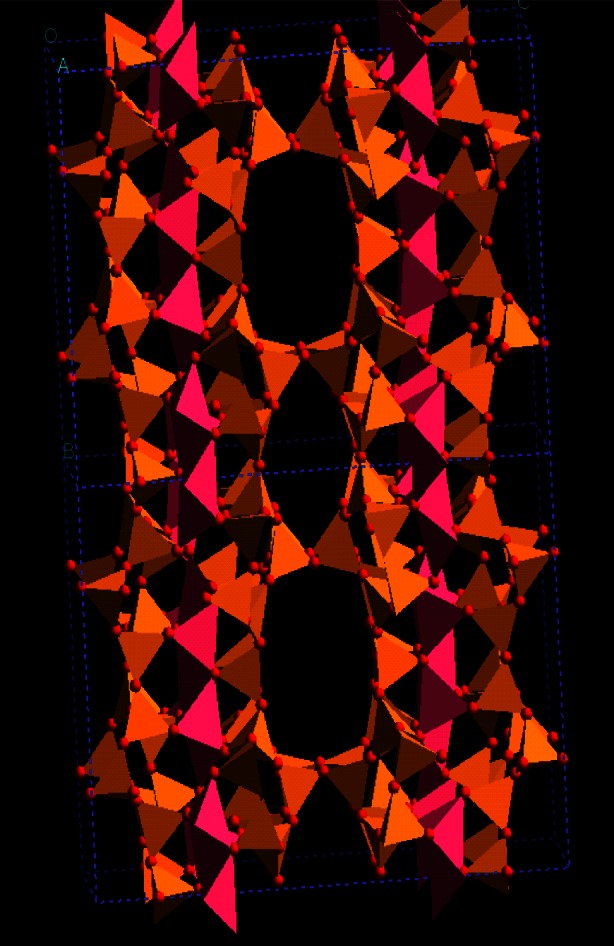
Framework geometry of litho silicate RUB-29. Oxygen atoms are shown as red spheres. The SiO_4_ units are shown as brown tetrahedra and LiO_4_ units as pink tetrahedra. Charge balancing cations, sited in the pores, are omitted for clarity.

There is great interest in finding new zeolitic frameworks, e.g., materials with new pore structures, since these materials have the potential to offer unique properties. For this reason there was considerable excitement accompanying the discovery that novel microporous materials can be prepared with frameworks composed of SiO_4_ and LiO_4_ tetrahedral [86, 87]. The LiO_4_ tetrahedral appear to be more flexible than SiO_4_ or AlO_4_ tetrahedral and thus these lithosilicate materials contain building units that would be highly strained in an aluminosilicate. The framework of one such material, RUB-29 is shown in Fig. 28. The approximate framework structure was determined using single-crystal synchrotron diffraction from an extremely small (≈200 µm^3^) crystal. Neutron diffraction was then used to determine the Li sitting. NMR measurements indicate that RUB-29 is also novel in that all Li atoms in this material appear to be mobile below 250 °C. This suggests that lithosilicates could be useful as ion conductors, perhaps in batteries or fuel cells.

## 5. Crystallography in Engineering Research

(This section was written by Thomas Gnäupel-Herold, NIST Center for Neutron Research)

### 5.1 Introduction

An important role of mechanical engineering is the calculation of safety margins and the lifetime prediction of parts and components. A key factor for these considerations is the knowledge of stresses a component would experience in service. While the fraction of external stresses is usually well-known, residual stresses can be problematic because they superimpose on the external stresses resulting in a total stress level that can exceed the strength of the material, thus causing unexpected failures. In addition to manufacturing induced residual stresses, there are also service-induced stresses originating from fatigue, corrosion and other processes that often determine the lifetime of an engineering component.

Due to the high penetration of neutrons in many materials, neutron diffraction residual stress analysis (NDRSA) is one of the few methods able to provide information about the three-dimensional strain and stress distribution at depths of several centimeters in engineering components nondestructively. In comparison, x rays from laboratory sources in the energy range 5 keV to 20 keV are limited to about 20 µm penetration. Thus, NDRSA can act as a tool for providing basic data for the design of a component, model validation of finite element calculations or even for quality control at certain stages of the life of a part. Recognizing these uses and the need for such a tool for American industry and academia, a collaborative program of research in this area was begun in the early 1980s. The NIST Center for Neutron Research began construction of a new, dedicated double axis system for residual stress, texture and single crystal analysis (DARTS) in1991. The instrument has been in operation successfully since fall 1995.

### 5.2 Principle of the Method

As diffraction is basically a length measurement, lattice spacing act as a strain gauge for a stress in a certain direction of the part or component. The relationship between the wavelength of the neutrons *λ*, the lattice spacing *d_hkl_* of the atomic planes described by the Miller indices *hkl*, and the diffraction angle is given by Bragg’s law.

$$\lambda =2d_{hkl}\;\sin\;\theta_{hkl}.$$ (13)

**Fig. 29:**
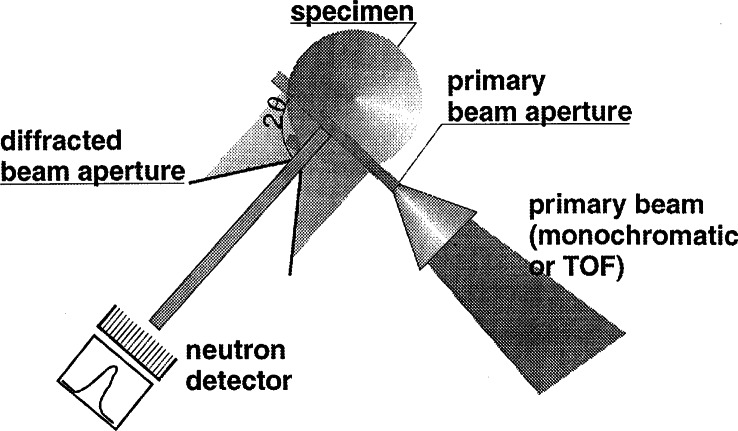
Schematic of diffraction geometry and slit arrangement.

The *d*-spacing information does not come from a point, it describes the volume average over a region called the sampling volume. The shape and dimensions of the sampling volume are prescribed by two slit systems. The first set of slits defines the incident beam which is diffracted throughout its entire path through the sample. The secondary slits in front of a neutron detector single out a narrow part of that diffracted beam. The intersection of both beams defines the sampling volume (see Fig. 29). Usually the wavelength is chosen so that *2θ_hkl_* is approximately 90° because then the cross-section of the sampling volume becomes rectangular. Then, its dimensions are the width of primary beam times the height times the horizontal opening of the receiving slit. The position of the sampling volume is constant. Three-dimensional mapping of strain in a component is accomplished by using an *x-y-z*-positioning table which can move any point in the component into the sampling volume.

If a reference value for the *d*-spacing can be found, e.g., by measuring *d*-spacings in a small coupon in which all long-range stresses are relieved, so that the calculation of strain from the measured *d*-spacings becomes straightforward

$$\varepsilon \left(hkl,\;\varphi ,\;\psi \right)=\frac{d_{hkl}-d_{hkl}^{0}}{d_{hkl}^{0}}.$$ (14)

The angles *φ* and *ψ* describe the orientation of the component (specimen) with respect to the reference frame. Using strain measurements in at least six independent directions the complete strain tensor can be obtained from

$$\varepsilon \left(hkl,\;\varphi ,\;\psi \right)=\sum_{ij}\varepsilon_{ij}n_{i}n_{j}$$ (15)

in which *n* = (cos*φ* sin*ψ*, *sinφ sineψ*, cos*ψ*). In order to obtain stress from strain, the elastic moduli in Hooke’s law have to replaced by the plane specific values *E_hkl_* and *v_hkl_* for translating *lattice* strain into *macroscopic* stress.

### 5.3 Instrumentation

A residual stress neutron diffractometer is nothing more than a specialized powder diffractometer. The differences are in the sophisticated slit assembly, the available wavelength range, the resolution and, most of all, an *x-y-z* -table for moving and positioning heavy specimens.

A residual stress measurement requires slit systems that define the sampling volume because this is the best way to assign the measured *d*-spacing to a point or, because it is not possible to measure a point, to a small region within the sample. Without these slits, the d-spacing would represent the spatial average over the entire illuminated region. Also, because of the large penetration of neutrons, this region would extend over several cm^3^ instead of a few mm^3^ as required by most problems. The available neutron flux usually makes sampling volumes <0.5 mm^3^ unfeasible.

Because of the requirement of having 2*θ_hkl_* around 90° for good spatial resolution there is no need for a large angular opening in the scattering plane. At NIST the detector is a linear position sensitive detector of 100 mm length which under normal conditions provides an opening of about 8°—enough to record a complete diffraction peak.

In order to bring almost any desired peak to 90° the instrument has a total of three different monochromators with six available reflections. Together with a variable monochromator diffraction angle *θ*_M_ the wavelength range is from 0.95 Å to 3.13 Å. There is also enough overlap between the wavelength range of each monochromator so that the optimal setting can be chosen that is given by the figure of merit (FM). The FM is, roughly speaking, the neutron flux at the sample position divided by the square of the standard deviation of the recorded diffraction peak. It is a measure of the counting time necessary to reach a certain for the chosen d-spacing. In fact, a residual stress machine is optimized for the best possible FM which makes it more of a medium resolution diffractometer. Unlike powder diffractometers which may be more tuned towards better resolution and make up for the loss of intensity by bigger samples, bigger beams, and multiple detectors, the design of a residual stress diffractometer is driven by the size of the sampling volume. The goal is to measure a strain with a given accuracy for a volume ≈ 30 mm^3^ or smaller in the shortest possible time.

**Fig. 30:**
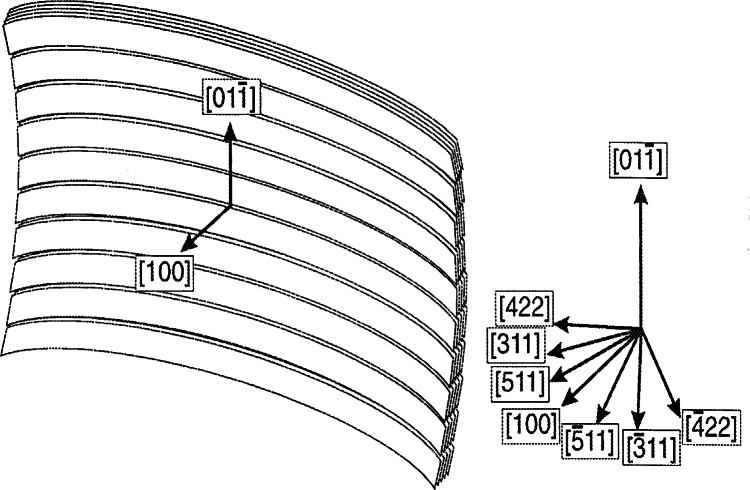
Schematic drawing of the double focusing perfect silicon monochromator On the right side the useful reflections of the device are shown.

Big improvements in the figure of merit were made possible by the recent development of perfect silicon double focusing monochromators. The concept is to utilize the large in-pile acceptance (area about 18 cm^2^×13 cm^2^) and to focus otherwise unused neutrons into a narrower spot at the sample position. The last generation of these devices utilizes packets of thin silicon wafer blades that are stacked vertically and tilted slightly with respect to one another so that the whole assembly has a fixed vertical curvature. Horizontally, i.e. within the scattering plane, the curvature is produced by elastic bending of the packets in a four-point bending device. Figure 30 shows a schematic diagram of the device used at NIST.

The optimal horizontal curvature, i.e., the curvature that produces the highest FM, depends on a variety of factors but the most important one is the monochromator diffraction angle *θ*_M_. Because the versatility of a variable *θ*_M_ is needed for changing the neutron wavelength it is also necessary to change the horizontal curvature to the respective optimal setting. Such a device is used at the DARTS diffractometer at the NCNR.

Aside from the instrumentation for delivery and detection of neutrons, there is also peripheral equipment. This includes a four-circle goniometer for single crystal and preferred orientation measurement, a load frame for the investigation of elastic and plastic deformation characteristics and heating devices for the measurement of stress at elevated temperatures.

### 5.4 Examples of Neutron Diffraction Residual Stress Measurements

**Fig. 31:**
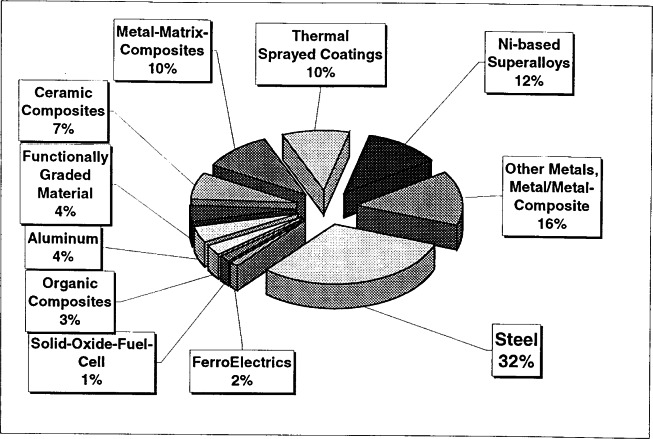
Materials investigated with residual stress diffractometers world wide (selected).

Most of the residual stress measurements are related in one way or another to industrial applications. Therefore the most frequently investigated samples are the technologically important materials such as steel, aluminum, nickel-alloys and ceramics. The diagram of Fig. 31 shows an overview of the materials that were investigated 1999 at the residual stress diffractometers at NIST, HIFR (Oak Ridge), ILL (France) and ISIS (UK).

NDRSA provides depth profiling capabilities at spatial resolutions that match both the requirements imposed by finite element meshing and by the characteristic length scale within which steep strain gradients are present in a sample. This way even detailed maps of three dimensional strain and stress distributions can be obtained.

**Fig. 32:**
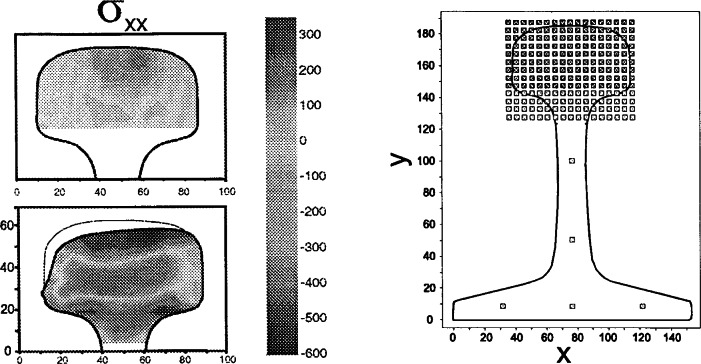
Residual stress distribution in an unknown (top) and a worn (bottom) rail. Red colors indicate tensile stresses, blue stands for compressive stress. Values on the color bar are given in megapascals. The right side shows the mesh of sampling volumes with size and location within the rail.

Examples for the work that has been done at the NIST Center for Neutron Research include welds in aluminum, steel and stainless steel; automotive springs; ceramics and metal-matrix composites; induction-hardened fatigue specimens; structural parts of aircrafts. A good example for the strain mapping capabilities of NDRSA is a project done in collaboration with the U.S. Department of Transportation and residual stresses in rails. The purpose of this investigation was to determine the effect of grinding strategies on the distribution and magnitude of service-induced residual stresses. Their importance relies on the fact that they are critical parameters in the estimation of the growth rate of fatigue defects. This information is used to assess rail inspection frequencies to assure defect detection prior to catastrophic rail failure. Figure 32 shows the two dimensional residual stress distribution a unworn and a worn rail obtained from a mesh of sampling volumes of 3 mm^3^×3 mm^3^×3 mm^3^ (right side).

The above example illustrates current capabilities of NDRSA. Stresses can be obtained from depths of about 3 cm in steel, 6 cm in aluminum and 2 cm in nickel using sampling volumes of 30 mm^3^ to 50 mm^3^. Further instrumental improvements will not increase that depth significantly because of the exponential attenuation of the neutron beam. The gains in the figure of merit will rather go into improved stress accuracy and smaller sampling volumes. For example, an improvement in the figure of merit by a factor of two enables a sampling volume half the previous size at the same counting time and stress accuracy.

## 6. Search for Novel Superconducting Materials

(This section was written by Q. Huang, NIST Center for Neutron Research)

As mentioned above, a lot of work has been done on the structural determinations of the high *T*_c_ superconducting materials using neutron powder diffraction. In particular, materials with the oxide perovskite type structure have attracted interest in recent years because this structure type seems to be an excellent structural framework for promoting superconductivity, eclipsing the intermetallic compounds which have been the source of many superconducting materials in the past. However, the recent discovery of superconductivity in MgB_2_ [88] suggests that intermetallic compounds with simple structure types are worth serious reconsideration as sources of new superconducting materials. This suggestion is corroborated by the discovery of superconductivity at 8 K in the intermetallic compound MgCNi_3_, which has the perovskite structure [9]. The high proportion of Ni in the compound suggests that magnetic interactions may be important to the existence of the superconductivity. MgCNi_3_, in fact is best considered as the three-dimensional analog of the layered nickel borocarbides, typified by LuNi_2_B_2_C, which has a *T*_c_ near 16 K [89]. The discovery also indicates that MgB_2_ and MgCNi_3_ will not be the only superconductors of this kind, and that a significant new class of superconducting materials may be found down this path in the future.

**Fig. 33:**
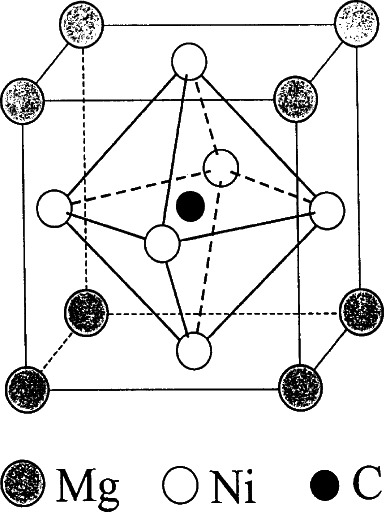
Schematic representation of the crystal structure of the 9 K superconductor MgCNi_3_.

Figure 33 shows the structure of the non-oxide perovskite intermetallic compound MgC*_x_* Ni_3_ which has a *Pm*3*m* symmetry with atomic positions of Mg: (0, 0, 0), C: (1/2, 1/2, 1/2), and Ni: (0, 1/2, 1/2). The C atom is surrounded by Ni atoms forming CNi_6_ octahedra which, in the oxide perovskites, are formed by a metallic atoms with six surrounding oxygen atoms. The magnetization data [89]show that the magnetic onset for the superconducting transition ranges between 7.1 K for a nominal carbon content of 1.1 per MgCXNi_3_, to 7.4 K for nominal carbon content *x* = 1.5. For nominal C contents between *x* = 1.1 and *x* = 1.0 the superconducting transition turns off abruptly. The C content *x* in the structure must play a major role for the superconductivity to exist.

The structure refinement, using the neutron powder diffraction data collected at BT1, reveals that the composition of the superconducting phase for nominal *x* = 1.25 (*T*_c_ = 7.3 K) is MgC_0.96_Ni_3_. This result is in agreement with the small amount of unreacted graphite (2 % by weight) found in the sample. Therefore the data suggest that the highest *T*_c_ in the perovskite is obtained for the stoichiometric composition MgC_1.0_Ni_3_. Further the data suggest that the superconductivity disappears abruptly for carbon contents between 0.96 and approximately 0.90 per formula unit.

**Fig. 34:**
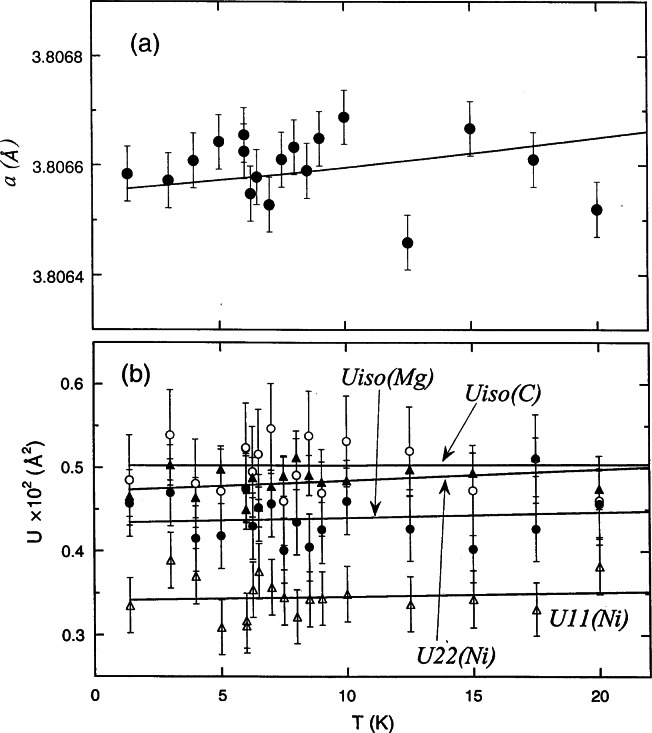
Plot of the *a* -parameter (top) and the thermal factors (bottom) as function of temperature, in MgCNi_3_. The behavior of these parameters shows no detectable anomalies at the critical temperature.

MgC_0_._96_Ni_3_ shows no magnetic or structural transitions between 2 K and 295 K [90]. Fitting with a Gausian function all the peaks above 70° 2-theta at different temperatures indicates that the peak width and shape are close to the instrumental resolution, i.e., no observable particle and/or strain broadening are present in the measured temperature range, and that no evidence of other structural distortions was found with decreasing temperature. The refined lattice parameter *a* and Debye-Waller factors decrease smoothly as the temperature decreases. Careful measurements near *T*_c_ (7.3 K) show, as shown in Fig. 34, that the changes of those parameters are within the experimental uncertainties, which are relatively small. Therefore if there is any structural anomaly at *T*_c_, it would be undetectable with the degree of precision of these measurements, which is of the order of 0.01 %.

## Figures and Tables

**Table 1:** Absorption in YBa_2_Cu_3_O_7_ (molecular weight: 666.5)

Elements	Atomic wt.	X-Ray (*λ* = 1.546) Å		Neutron (*λ* = 1.08) Å	

		µ/ρ^a^	*µ/ργ*^b^	µ/ρ^a^	*µ/ργ*
Y	88.9 × 1	134	17.9	0.006	0.001
2Ba	137.4 × 2	330	136.1	0.003	0.001
3Cu	63.6 × 3	52.9	15.2	0.002	0.001
7O	16.0 × 7	11.5	1.9	0.000	0.000
Σ	666.5		171.0		0.003

a cm^2^ g^−1^ [23]

b *γ* is the mass fraction of the corresponding chemical element.

**Table 2:** Comparison of Rietveld refinements for *γ*Ba_2_Cu_3_O_7_ obtained in four laboratories

Atom	Parameter	Ref. [55]	Ref. [56]	Ref. [57]	Ref. [20]
Ba	*z*	0.1839(3)	0.1841(3)	0.1843(3)	0.1839(2)
	*B*	0.4(1)	0.6(1)	0.54(5)	0.65(5)
Y	*B*	0.2(1)	0.6(1)	0.46(4)	0.56(4)
Cu(1)	*B*	0.2(1)	0.4(1)	0.50(5)	0.55(4)
Cu(2)	*z*	0.3546(2)	0.3549(3)	0.3556(1)	0.3547(1)
	*B*	0.3(1)	0.5(1)	0.29(4)	0.49(4)
O(1)	*z*	0.1589(3)	0.1581(4)	0.1584(2)	0.1581(2)
	*B*	0.5(2)	0.9(1)	0.67(5)	0.78(5)
	*n*	2.01(3)	2.0	2.0	2.0
O(2)	*z*	0.3783(3)	0.3779(4)	0.3773(2)	0.3779(2)
	*B*	0.5(2)	0.1(1)	0.56(5)	0.57(5)
	*n*	2.01(3)	2.0	1.89(2)	2.0
O(3)	*z*	0.3780(3)	0.3777(5)	0.3789(3)	0.3776(2)
	*B*	0.4(2)	0.3(1)	0.37(5)	0.55(5)
	*n*	2.02(3)	2.0	2.0	2.0
O(4)	*B*	1.6(3)	2.4(3)^a^	1.35(5)	1.73(9)
	*n*	0.96(2)	1.0	0.92(2)	1.0
O(5)	*B*	1.6			
	*n*	0.04(1)			
	a	3.8172(1)	3.8206(1)	3.8231(1)	3.8198(1)
	b	3.8822(1)	3.8851(1)	3.8863(1)	3.8849(1)
	c	11.6707(4)	11.6757(4)	11.6809(2)	11.6762(3)

a Equivalent isotropic *B* from anisotropic results.

**Table 3:** Composition and *T*_c_ values in the homologous series HgBa_2_Ca*_n_*_−1_O*_n+2+x_*

Compound		*x*	*T*_c_
HgBa_2_Ca_5_Cu_6_O_14+_*_x_*	1256	0.065	126–128 K
HgBa_2_CuO_4+_*_x_*	1201	0.065	94–95 K
HgBa_2_CaCu_2_O_6+_*_x_*	1212	0.22	126–128 K
HgBa_2_Ca_2_Cu_3_O_8+_*_x_*	1223	0.41	133 K
HgBa_2_Ca_5_Cu_6_O_14+_*_x_*	1256	0.40	114 K

## Footnotes

1 Certain commercial equipment, instruments, or materials are identified in this paper to foster understanding. Such identification does not imply recommendation or endorsement by the National Institute of Standards and Technology, nor does it imply that the materials or equipment identified are necessarily the best available for the purpose.
